# Systematic review and meta-analysis of diagnostic accuracy of detection of any level of diabetic retinopathy using digital retinal imaging

**DOI:** 10.1186/s13643-018-0846-y

**Published:** 2018-11-07

**Authors:** Mapa Mudiyanselage Prabhath Nishantha Piyasena, Gudlavalleti Venkata S. Murthy, Jennifer L. Y. Yip, Clare Gilbert, Tunde Peto, Iris Gordon, Suwin Hewage, Sureshkumar Kamalakannan

**Affiliations:** 1 Clinical Research Department, International Centre for Eye Health, London School of Hygiene and Tropical Medicine, Keppel Street, London, WC1E 7HT UK; 2 School of Medicine, Dentistry and Biomedical Sciences, Queen’s University, 97, Lisburn Road, Belfast, BT9 7BL Northern Ireland; 3 Retina Research Unit, National Eye Hospital, Deans Road, Colombo, 01000 Sri Lanka; 4 Indian Institute of Public Health, Plot No 1 Kavuri Hills Madhapur, Hyderabad, 500033 India

**Keywords:** Diabetes mellitus, Diabetic retinopathy, Diagnostic test accuracy, Digital imaging, Mydriatic, Non-mydriatic, Low income, Screening

## Abstract

**Background:**

Visual impairment from diabetic retinopathy (DR) is an increasing global public health concern, which is preventable with screening and early treatment. Digital retinal imaging has become a preferred choice as it enables higher coverage of screening. The aim of this review is to evaluate how different characteristics of the DR screening (DRS) test impact on diagnostic test accuracy (DTA) and its relevance to a low-income setting.

**Methods:**

We conducted a systematic literature search to identify clinic-based studies on DRS using digital retinal imaging of people with DM (PwDM). Summary estimates of different sub-groups were calculated using DTA values weighted according to the sample size. The DTA of each screening method was derived after exclusion of ungradable images and considering the eye as the unit of analysis. The meta-analysis included studies which measured DTA of detecting any level of DR. We also examined the effect on detection from using different combinations of retinal fields, pupil status, index test graders and setting.

**Results:**

Six thousand six hundred forty-six titles and abstracts were retrieved, and data were extracted from 122 potentially eligible full reports. Twenty-six studies were included in the review, and 21 studies, mostly from high-income settings (18/21, 85.7%), were included in the meta-analysis. The highest sensitivity was observed in the mydriatic greater than two field strategy (92%, 95% CI 90–94%). The highest specificity was observed in greater than two field methods (94%, 95% CI 93–96%) where mydriasis did not affect specificity. Overall, there was no difference in sensitivity between non-mydriatic and mydriatic methods (86%, 95% CI 85–87) after exclusion of ungradable images. The highest DTA (sensitivity 90%, 95% CI 88–91%; specificity 95%, 95% CI 94–96%) was observed when screening was delivered at secondary/tertiary level clinics.

**Conclusions:**

Non-mydriatic two-field strategy could be a more pragmatic approach in starting DRS programmes for facility-based PwDM in low-income settings, with dilatation of the pupils of those who have ungradable images. There was insufficient evidence in primary studies to draw firm conclusions on how graders’ background influences DTA. Conducting more context-specific DRS validation studies in low-income and non-ophthalmic settings can be recommended.

**Electronic supplementary material:**

The online version of this article (10.1186/s13643-018-0846-y) contains supplementary material, which is available to authorized users.

## Background

Diabetes mellitus (DM) is one of the most prevalent non-communicable diseases and has significant impacts on health systems [1]. The International Diabetes Federation (IDF) estimated that there were 425 million people with DM (PwDM) in the world in 2017 which is projected to increase to 629 million by 2045 [2]. The greatest impact affects low- and middle-income countries (LMIC) (overall increase 69%) due to ageing population, obesity and sedentary life style [3]. This is exacerbated by weak health systems coupled with slow economic development [4]. Diabetic retinopathy (DR) is a common microvascular complication of DM caused by chronic hyperglycaemia [5]. A pooled meta-analysis using population-based studies conducted in the USA, Australia, Europe and Asia showed that the prevalence of any DR in PwDM aged 20 to 70 years was 34.6% (95% CI 34.5–34.8%): proliferative DR affected 6.96% (95% CI 6.87–7.04%) and sight-threatening DR (STDR) affected 10.2% (95% CI 10.1–10.3%), globally translating to approximately 28 million PwDM affected by STDR [6]. DR is a leading cause of blindness among the young and middle-aged adults in most of the high-income countries (HIC).

Many studies have shown that control of risk factors, early DR screening (DRS) and appropriate treatment can reduce the risk of blindness and visual impairment due to DR [7–12]. Digital retinal imaging has been widely practiced and an accurate method for DRS [13]. Providing appropriate training to photographers is of paramount importance, and with enough practice, high levels of competence can be achieved by those taking imaging regularly. Non-mydriatic digital imaging methods cause less discomfort and are more convenient for service providers. However, poor image quality is an important limitation of digital retinal imaging, particularly if non-mydriatic systems are being used, in countries where cataract is common [14].

In current literature, a systematic review showed that dilated imaging aided by fundoscopy for ungradable images was an effective modality to screen for DR [15]. This review included studies from 1985 to 1998 when digital retinal imaging technology was not available. Shi et al. concluded that accuracy of detecting presence/absence of DR by tele-medicine using digital imaging is high (pooled sensitivity 80%, 95% CI 84–88%; pooled specificity 89%, 95% CI 88–91%) [16]. Another meta-analysis concluded that dilatation of the pupils did not have a bearing on the diagnostic test accuracy (DTA) for any level of DR (sensitivity: odds ratio (OR) − 0.89, 95% CI 0.56–1.41, *p* = 0.61; specificity: OR 0.94, 95% CI 0.57–1.54, *p* = 0.80) [17]. A limitation of this review was that results from different imaging methods (i.e. polaroid, film and digital) and clinical examination were pooled into one estimate.

A DRS modality which is suited to the health system and its context is a key factor in the success of a programme [18]. A screening programme requires substantial investment in infrastructure and workforce development. LMICs have low capacity to implement a population-based DRS programme (DRSP) with routine call/recall and full DR patient list. Yet there is a high burden of unmet need, with higher levels of uncontrolled DM leading to higher rates of DR progression. Weak health systems require a DRSP where detection of any DR using most effective and efficient instruments would be most useful. In addition, resources are scarce, and so efficient use of both equipment and human resources are essential. The detection of clinic-based PwDM with any DR will enable identification and stratifying risk groups early and screen safely at a lower threshold at non-ophthalmic settings. Therefore, a feasible way of providing accessible services is to offer digital photographic DRS when PwDM present for routine medical care at diabetologist/physicians’ clinics. In a low-income setting, identification of a person with any DR/no DR would be a helpful stratification for the providers. In a practical programme guideline, we would suggest performing mydriatic imaging or refer to the next level for those with ungradable images. There is also a lack of understanding among the PwDM about the benefits of mydriasis. Discomfort experienced after pupil dilatation has led to low uptake in dilated examination [19]. Therefore, it is important to understand the best method to detect any DR in non-specialist settings that will be suited to LMICs [18].

The objectives of this review were to evaluate how using or not using pharmacological dilation of the pupil and the number of fields captured influence DTA and how well different ophthalmic and non-ophthalmologist health care professionals perform DR grading compared to seven-field image grading or mydriatic ophthalmoscopy by ophthalmologists in different clinical settings. This will inform decision-making for choosing strategy in those aspects of a DRSP. This is an assessment of accuracy of instruments for a systematic clinic-based screening rather than a population-based screening tool. We plan to propose most efficient modality for provision of DRS to PwDM at non-ophthalmic settings (i.e., medical clinic, endocrinology clinic) using this evidence.

## Methods

The Preferred Reporting Items for Systematic Reviews and Meta-Analysis (PRISMA) guidelines were followed in reporting (The PRISMA checklist is available as Additional file 1).

### Eligibility criteria and study context

We included studies of cross-sectional study designs that aimed to evaluate the accuracy of DRS using digital imaging as the index test, in PwDM at permanent healthcare facilities. We used the Early Treatment Diabetic Retinopathy Study (ETDRS) seven-field image interpretation as the gold standard and mydriatic bio-microscopy/ophthalmoscopy by an ophthalmologist/retinologist as the clinical reference standard where the gold standard was not performed. The primary context considered for this review was institutional DRS clinics/programmes using digital imaging. We categorised the context as either primary or secondary/tertiary. We excluded studies conducted in informal health facilities, used automated analysis systems, used non-digital imaging methods in index test, used mobile screening methods or did not report on DTA as an outcome measure.

### Primary outcome

The outcome examined was sensitivity and specificity of detection of ‘any level of DR’. It is important to understand the optimal method to detect any DR in non-specialist settings, especially in LMICs where PwDM have higher risk of progression, due to poorly controlled risk factors and irregular follow up. ‘Any level’ of DR was considered appropriate as we felt that such an approach would have collateral benefits like raising awareness among the providers as well as augmenting awareness of PwDM regarding the importance of regular follow-up and control of the risk factors minimising the progression to STDR.

### Search and study selection

**Fig. 1:**
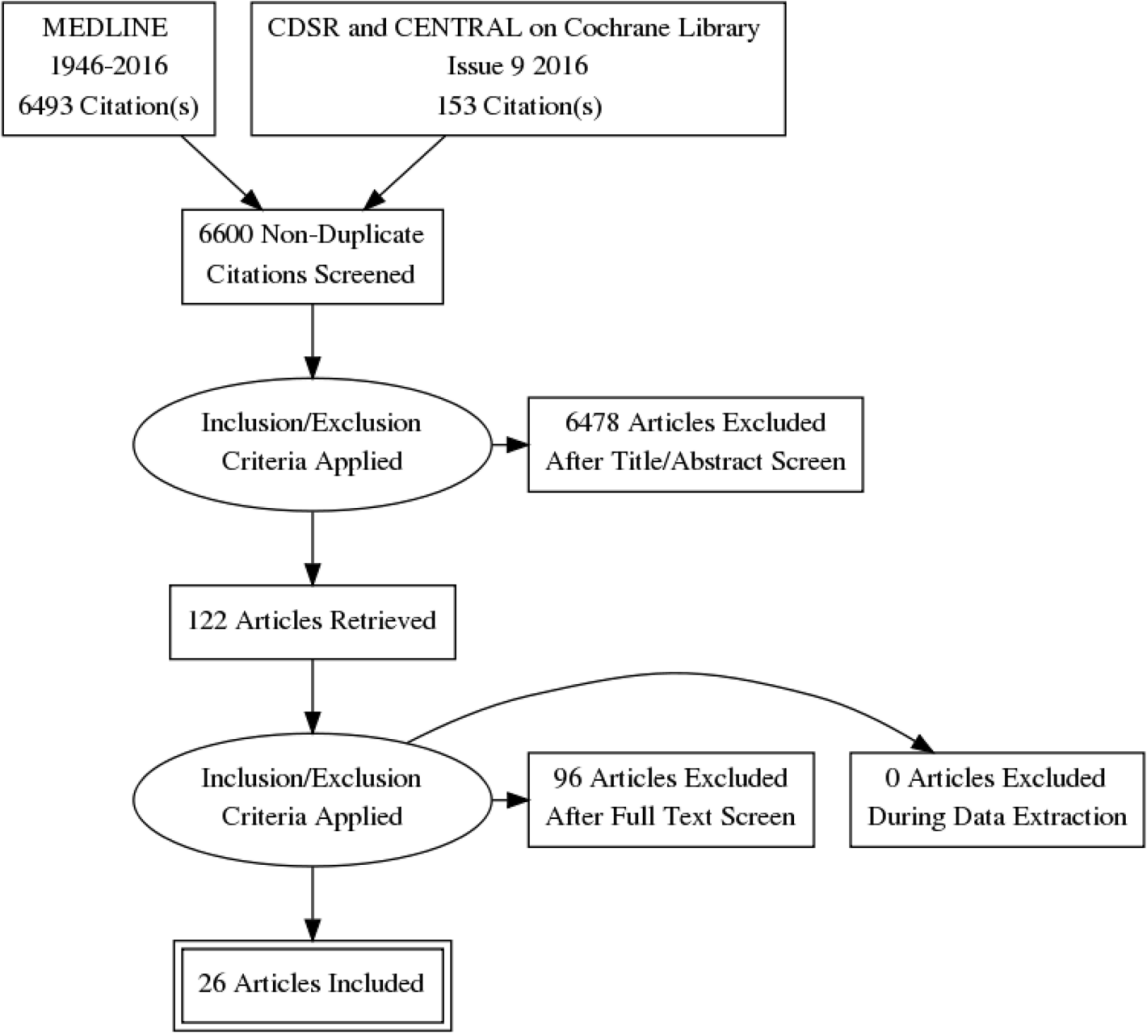
PRISMA flow diagram of the study selection process

We developed a comprehensive search strategy to obtain published articles by consulting an information specialist and searched MEDLINE (Ovid), Cochrane Database of Systematic reviews (CDSR) and CENTRAL in the Cochrane Library. The databases were searched from the date of inception of the databases to September 2016, to identify any published reviews on this topic and to see whether relevant trials where included in the CENTRAL database. The search terms and strategy are shown in Table 1 and Fig. 1 respectively. Two reviewers (PN and SK) independently assessed the eligibility of the titles and abstracts, and discrepancies were solved by consulting a third reviewer (GV). Full papers of the eligible articles (*n* = 122) were obtained from the publishers/authors.

**Table 1:** Electronic database search terms

1. Exp mass screening/ 2. Exp vision tests/ 3. Exp telemedicine/ 4. Exp Photography/ 5. Exp ophthalmoscopes/ 6. Exp ophthalmoscopy/ 7. (ophthalmoscop$ or fundoscop$ or funduscop$) . ti . 8. ((photo$ or imag$) adj3 fundus) . tw . 9. (Photography or retinopgraphy) . tw . 10. ((mydiatric or digital or retina$ or funduc or stereoscopic) adj3 camera) . tw . 11. ((mydiatric or digital or retina$ or fundus or stereoscopic) adj3 imag$) .tw . 12. Screen$ . tw .	13. ((eye$ or retina$ or ophthalm$) adj4 exam$).tw . 14. ((eye$ or vision or ophthalmic) adj4 test$) . tw. 15. ((eye$ or retina$ or ophthalm$) adj4 visit$) . tw . 16. Office visits/ 17. (telemedicine$ or telemonitor$ or telescreen$) . tw . 18. 1 or 2 or 3 or 4 or 5 or 6 or 7 or 8 or 9 or 10 or 11 or 12 or 13 or 14 or 15 or 16 19. exp diabetes mellitus/ 20. exp diabetes complications/ 21. 19 or 20 22. exp diabetic retinopathy/ 23. ((diabet$ or proliferative or non-proliferative) adj4 retinopath$).tw. 24. 21 or 22 25. 18 and 21 and 24

### Data collection process

A data extraction form was prepared, and data were extracted and entered into a formatted MS Excel® database. Data from all the full reports of filtered citations (*n* = 122) were extracted. We used a modified Strengthening the Reporting of Observational Studies in Epidemiology (STROBE) guidelines for cross-sectional studies to identify the components to extract [20]. The modifications made were based on Cochrane guidelines on conducting systematic reviews of studies of DTA [21]. Two independent reviewers extracted the data (PN and SK) from full reports. In the piloting stage, data were extracted from 10% (12/122) of the articles by two reviewers and consistency was checked (SH). Corrections to the data extraction sheets and databases were done at this stage. The data extracted of all the included articles (*n* = 26) were checked by the co-reviewer (SK) for consistency.

### Data items

The data extracted from each study included country, study design, study setting, sample size and participant characteristics (mean age with standard deviation and range, male to female ratio, number of years with DM). The next section of the extraction included study objectives, sampling strategy, methods of index test (degree of view, number of fields, pupil status and type of camera) and method of reference standard. Finally, data on DTA (sensitivity with 95% CI, specificity with 95% CI, number of true positives, true negatives, false positives and false negatives, kappa value and gradability) were extracted. Studies were categorised according to the status of pupils, number of fields in imaging, type of index test grader and type of reference standard.

### Meta-analysis

Meta-analysis of the data was conducted to examine differences in outcome due to pupil status (mydriatic and non-mydriatic), number of retinal fields (one field, two fields, greater than two fields), type of index test grader (ophthalmologist, retinologist, retinal reader, ophthalmic registrars) and by the context (primary and secondary/tertiary). A sub-group meta-analysis was undertaken to determine the DTA of ‘any level’ of DR by non-ophthalmic personnel. Further sub-analyses were conducted by considering the studies which reported on DTA using the same participant imaged before and after pupil dilatation.

### Risk of bias in individual studies

We assessed the variations in bias using the Quality assessment of diagnostic accuracy studies - 2nd version (QUADAS-2) framework [22]. The methodological quality and applicability of the studies was considered using signalling questions under the four domains of patient selection, index test, reference standard and flow and timing [22]. We examined the differences in reported DTA estimates based on QUADAS-2 quality assessment guidelines, and given results in the meta-analysis were based on the studies identified to have low risk of bias. The methodological quality of the studies included in the review and meta-analysis are described in Table 2. All included studies were cross sectional in design as these demonstrated less bias in the QUADAS assessment. We considered the signalling questions according to the QUADAS-2 guidelines as examples, masking of the graders, inclusion of range of spectrum to reduce the spectrum bias, all participant undertaking all tests etc. when assessing the bias.

**Table 2:** Methodological quality and applicability assessment of the included studies (using QUADAS-2 guidelines)

Domains	Risk of bias				Applicability concerns		
Study	Patient selection	Index test	Reference standard	Flow and timing	Patient selection	Index test	Reference standard
1. Ahmed, J. et al. 2006	Low	Low	Low	High	High	Low	Unclear
2. Aptel, F. et al. 2008	Low	Low	Low	Unclear	Low	Low	Low
3. Baeza, M. et al. 2009	High	Low	Low	Low	Low	Low	Low
4. Boucher, M. C. et al. 2003	Low	Low	Low	High	High	High	Low
5. Ding, J. et al. 2012	Low	Low	Low	Low	Low	High	Low
6. Hansen, A. B. et al. 2004	High	Low	Low	Low	Low	Low	Low
7. Henricsson, M. et al. 2000	Low	Low	Low	Unclear	Low	Low	Low
8. Herbert, H. M. et al. 2003	Low	Low	Low	Low	Low	High	Low
9. Ku, J. J. et al. 2013	Low	Low	Low	Unclear	High	Low	Low
10. Kuo, H. K. et al. 2005	Low	Low	Low	Low	High	Low	Low
11. LopezBastida, J. et al. 2007	Low	Low	Low	Unclear	Low	Unclear	Low
12. Maberley, D. et al. 2002	Low	Low	Low	Low	High	High	Low
13. Massin, P. et al. 2003	Unclear	Unclear	Low	Low	High	High	Low
14. Murgatroyd H et al. 2003	Low	Low	Low	Low	Low	Low	Low
15. Neubauer, A. S. et al. 2008	Low	Low	Low	Unclear	Low	Low	Low
16. Olson, J. A. et al. 2003	Unclear	Low	Low	Unclear	Low	Low	Low
17. Phiri, R. et al. 2006	Unclear	Low	Low	Low	Low	High	Low
18. Scanlon, P. H. et al. 2003	Low	Low	Low	Low	High	High	Low
19. Scanlon, P. H. et al. 2003_(2)	Low	Low	Low	Low	Low	High	Low
20. Suansilpong, A et al. 2008	Low	Low	Low	Low	Low	Low	Low
21. Sundling, V. et al. 2013	Low	Low	Low	Low	Low	Low	Low
Studies excluded in meta-analysis							
22. Bhargava, M. et al. 2012	High	High	High	Unclear	Low	High	Low
23. Mizrachi, Y. et al. 2014	High	High	High	High	High	High	Low
24. Perrier, M. et al. 2003	High	Low	Low	High	High	High	Low
25. Schiffman, R.M. et al. 2005	Low	High	High	Unclear	Low	High	High
26. Tu, K.L. et al. 2004	High	High	High	Low	Low	High	Low

### Synthesis of results

Meta-analysis was conducted using STATA/IC (version-14.1, 2015-Texas-77845-USA) after acquiring the 2 × 2 table (TP, FP, TN and FN) values for number of eyes screened as the unit of analysis in each method of DRS. These values were cross checked by the number of DR positives and negatives reported in classification of findings under different categories of DR. The meta-analysis was conducted using the DTA of any DR, after excluding the ungradable images. Sub-analyses were conducted using the estimates that reported DTA on same participant groups before and after pupil dilatation and by non-ophthalmic index test graders.

Heterogeneity was assessed between the studies and between different modalities in the same study. Due to differences in definitions of the ungradable image category, we decided to exclude all ungradable images to minimise heterogeneity. At a practical programme level, all PwDM with ungradable images will be referred to the ophthalmologist’s clinic for further assessment. However, in this study, we were interested in the accuracy of the intervention to detect any DR, rather than any referable PwDM in a programme model.

## Results

The electronic database search yielded 6646 titles and abstracts, and 122 studies were selected to review full reports. Twenty-six studies were included in the review (Fig. 1). The details of the excluded articles are available as Additional file 2. We included 26 cross-sectional studies, and 88% (23/26) were conducted in HICs [23–45]. The remaining studies (3/26, 11%) were conducted in South East Asian upper middle-income countries (Thailand (one) [46], China (one) [47] and Taiwan (one) [48]). There were 6 studies (10 estimates) which reported DTA in which the same participant underwent imaging before and after pupil dilatation [25, 35, 40, 42, 44, 47].

The mean sample size of the studies was 316 PwDM screened (SE± 72.3, 95% CI 166–467, range 51–1549). Thirty percent (8/26) of studies selected participants from local and regional primary care units. Other studies recruited PwDM from retinal care (5/26, 19.2%), diabetes care (4/26, 15.3%), existing DR screening programmes (4/26, 15.3%), medical and ophthalmology care (1/26, 3.8%), retinal and ophthalmology care (1/26, 3.8%), ophthalmology care (1/26, 3.8%) and private sector optometry network (1/26, 3.8%). One study did not report the setting (1/26, 3.8%). The mean age of participants was 57.4 years (SE± .52, 95% CI 54.3–60.7, range 16–89 years): the mean age of participants in non-mydriatic strategies 58.9 years and mydriatic 59.0 years. The mean duration of known diabetes among participants was 12.0 years (SE ± 1.5, 95% CI 8.8–15.3 years), and 50.5% were male (SE ± 2.7, 95% CI 44.8–56.3). Participants’ characteristic tables of the studies included in this review are available as Additional file 3.

### Meta-analysis

Of these 26 studies, 5 studies (5/26, 19.2%) were not eligible for the meta-analysis. Those were excluded from the meta-analysis due to unavailability of required 2 × 2 table data, very high level of bias and heterogeneity. The study conducted by Perrier et al. used the same participants as in the study by Boucher et al. which has been included and another study was excluded due to a high likelihood of bias [33, 36]. The study conducted by Schiffman et al. was excluded as index test pupil status and number of retinal fields were not mentioned [30]. Two further studies were excluded: one only reported DTA for STDR [41] and another from Singapore (Bhargava et al.) did not provide DTA data [34].

Among 21 studies included in the meta-analysis, 39 different modalities were identified in terms of pupil status, retinal field strategy and human resources involved in index test DR grading. Forty-six percent (18/39 modalities) of the studies used non-mydriatic methods (13/21 studies) [25, 26, 29, 31, 35, 38, 40, 42, 44, 45, 47–49], 44% (17/39 modalities) used mydriatic methods (11/21 studies) [23–25, 32, 35, 37, 39, 40, 42, 44, 47] and ophthalmic personnel currently trained and practiced in DR grading had performed index test grading in these studies. In 10%, 4/21 [27, 28, 46, 48] newer non-ophthalmologist personnel had performed index test grading. Six studies reported mydriatic and non-mydriatic methods (6/21) [25, 35, 40, 42, 44, 47]. One study reported DTA values for ophthalmic and non-ophthalmic personnel [48]. The DTA of each screening strategy is available in Additional file 4.

### Studies included in secondary output analysis

Four studies were eligible for secondary output of meta-analysis of DTA of DRS as they used different non-ophthalmologist personnel [27, 28, 46, 48]. However, there were no adequate number of studies to meta-analyse by pupil status and field strategy. The details of these studies are described in Additional file 3 (participants’ characteristics) and Additional file 4 (DTA).

### Risk of bias and applicability concerns within studies

**Fig. 2:**
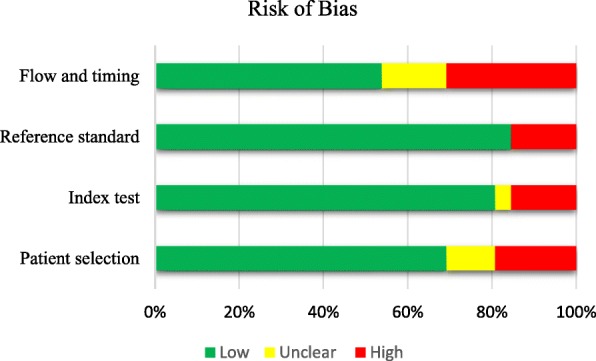
Proportion of included studies with a risk of bias

**Fig. 3:**
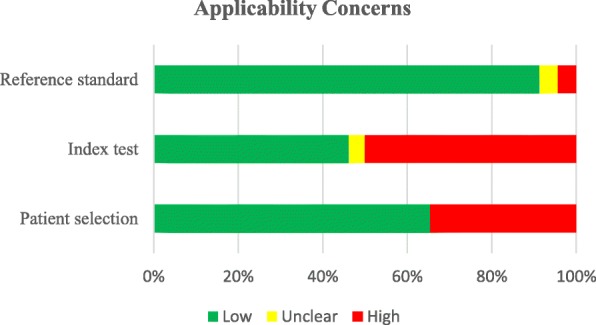
Proportion of included studies with applicability concerns

The methodological quality and applicability assessment of the included studies (Table 2) were according to the QUADAS-version 2 guidelines. In the assessment of bias, it was minimal (15.38% high risk) in conducting the index tests and reference tests. Nineteen percent of the studies showed high risk of bias in selection and 30.7% in participant flow and timing (Fig. 2). In the assessment of applicability, risk was minimal in reference standard (3.8%) and 34% of the studies showed high risk in applicability with regard to patient selection and 50% in index test (Fig. 3).

### Risk of bias in the included studies

There was selection bias in some studies: Baeza et al. excluded patients who had visited an ophthalmologist within 6 months of screening and those with hyper-mature cataract [44] and Boucher et al. purposively selected participants who had a greater risk DR [31]. There were also applicability concerns when authors reported the DTA of referable level of DR [38–40, 43, 47]. The study conducted by Hansen et al., which selected people with diabetes through a record review, was weighted towards less severe retinopathy, as mentioned by the authors [25]. Two studies attempted non-mydriatic methods and ended up dilating the pupils due to high proportion of ungradable images [23, 32]. In the study by Lopez-Bastida et al., the time interval between the index and reference tests was not stated, nor whether participants with ungradable images (90/773, 10%) underwent mydriasis while performing the index test [45]. Similarly, time and flow was not mentioned in the study by Ku et al. [37]. Two studies selected indigenous populations which lead to generalizability concerns [32, 37]. Furthermore, some studies were conducted in eye/retinal clinics where there was a possibility of high prevalence of advanced DR [39, 43, 48].

Reporting of DR was not uniform. In several studies, DTAs were reported for different levels of DR leading to some heterogeneity [25, 26, 31, 38–40, 43]. In these studies, we considered results for the detection of any level of DR. For example, Phiri et al. had defined DR including the macular signs which other authors had not considered and which would have an impact on the analysis [38].

### Diagnostic test accuracy in non-mydriatic imaging

**Fig. 4:**
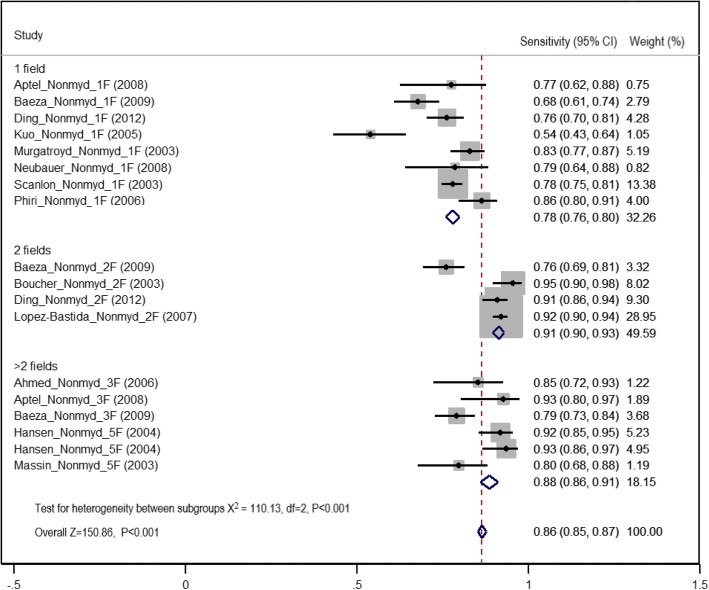
Forest plot of summary estimates of sensitivity of non-mydriatic imaging using different field strategies (1: one field, 2: two fields, 3: greater than two fields)

**Fig. 5:**
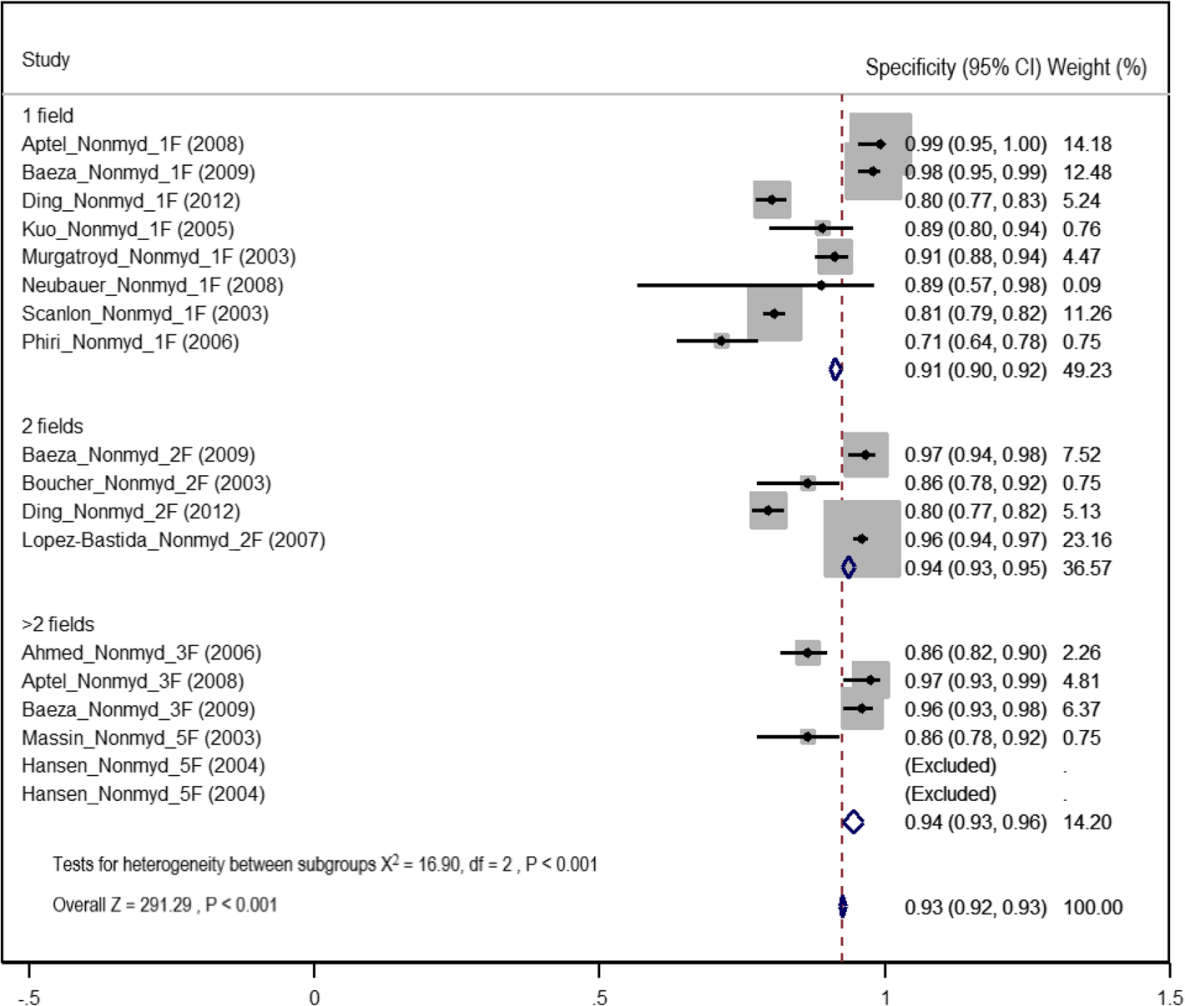
Forest plot of summary estimates of specificity of non-mydriatic imaging using different field strategies (1: one field, 2: two fields, 3: greater than two fields)

Among the 21 studies included in the meta-analysis, 18 used the following non-mydriatic imaging strategies: one field (8/18, 44.4%), two fields (4/18, 22.2%) and greater than two fields (6/18, 22.2%). The pooled sensitivity of detection of any level of DR using non-mydriatic digital imaging was 86% (95% CI 85–87%). The two-field strategy gave the highest estimate of sensitivity of 91% (95% CI 90–93%). The one and greater than two field strategies gave summary estimates of sensitivity of 78% (95% CI 76–80%) and 88% (95% CI 86–91%), respectively (Fig. 4, Table 3). The mean proportion of ungradable images in non-mydriatic methods was 18.4% (SE ± 2.2, 95% CI 13.6–23.3%). The summary estimate of specificity of detection of any level of DR using non-mydriatic digital imaging was highest in the two-field and greater than two field strategies (94%, two field 95% CI 93–95%, greater than two field 95% CI 93–96%). The one-field strategy gave pooled specificity values of 91% (95% CI 90–92%) (Fig. 5 and Table 3).

**Table 3:** Summary estimates of diagnostic test accuracy by field strategy and variations by reference standard

Imaging strategy		Non-mydriatic				Mydriatic			
		Reference—7F ETDRS^b^		Reference—DF slit lamp examination		Reference—7F ETDRS		Reference—DF slit lamp examination	
		Sensitivity (95% CI)	Specificity (95% CI)	Sensitivity (95% CI)	Specificity (95% CI)	Sensitivity (95% CI)	Specificity (95% CI)	Sensitivity (95% CI)	Specificity (95% CI)
Overall estimate		87%	96%	86%	91%	86%	96%	86%	87%
		(85–89%)^a^ (8)	(95–97) (6)	(85–88%) (10)	(91–92%) (10)	(84–89%) (5)	(95–97%) (5)	(85–87%) (12)	(86–88%) (12)
Field strategy	1F 1 field	79%	96%	78%	89%	77%	96%	80%	91%
		(74–83%) (2)	(95–98%) (2)	(75–80%) (6)	(88–90%) (6)	(70–82%) (1)	(95–99%) (1)	(78–83%) (6)	(90–92%) (5)
	2F 2 field	90%	96%	92%	93%	83%	95%	86%	75%
		(86–93%) (2)	(94–98%) (2)	(90–93%) (2)	(92–94%) (2)	(80–87%) (2)	(93–97%) (2)	(84–88%) (4)	(74–77%) (4)
	> 2F > 2 field	88%	95%	90%	94%	91%	93%	93%	95%
		(85–91%) (4)	(93–97%) (4)	(83–96%) (2)	(92–96%) (2)	(88–94%) (2)	(90–96%) (2)	(90–95%) (2)	(93–97%) (2)

*DF* dilated fundoscopy, *CI c*onfidence intervals

^a^Number of studies included in each estimate in meta

^b^7F ETDRS—early treatment diabetic retinopathy study seven-field strategy

### Diagnostic test accuracy in mydriatic imaging

**Fig. 6:**
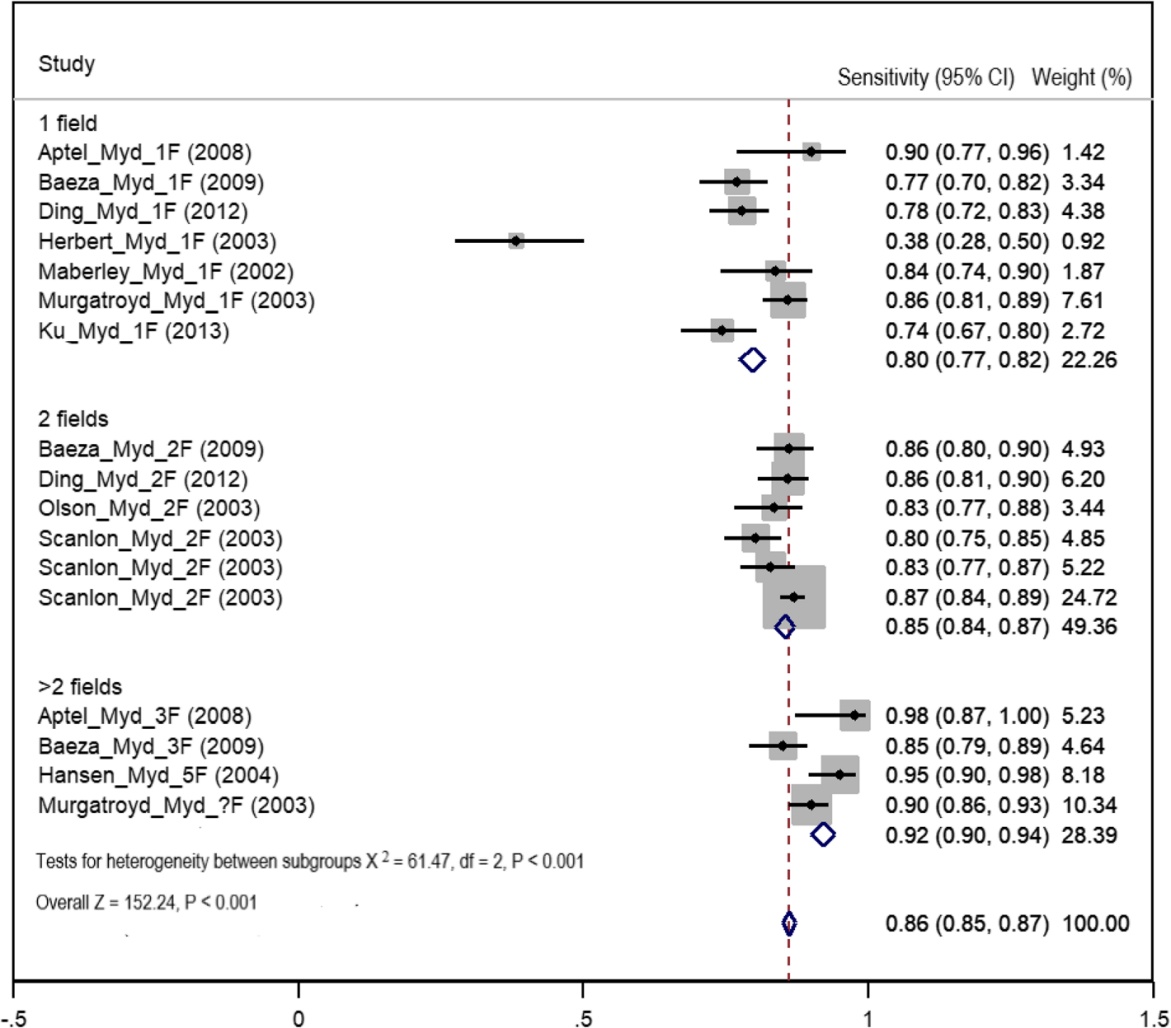
Forest plot of summary estimates of sensitivity of mydriatic imaging using different field strategies (1: one field, 2: two fields, 3: greater than two fields)

**Fig. 7:**
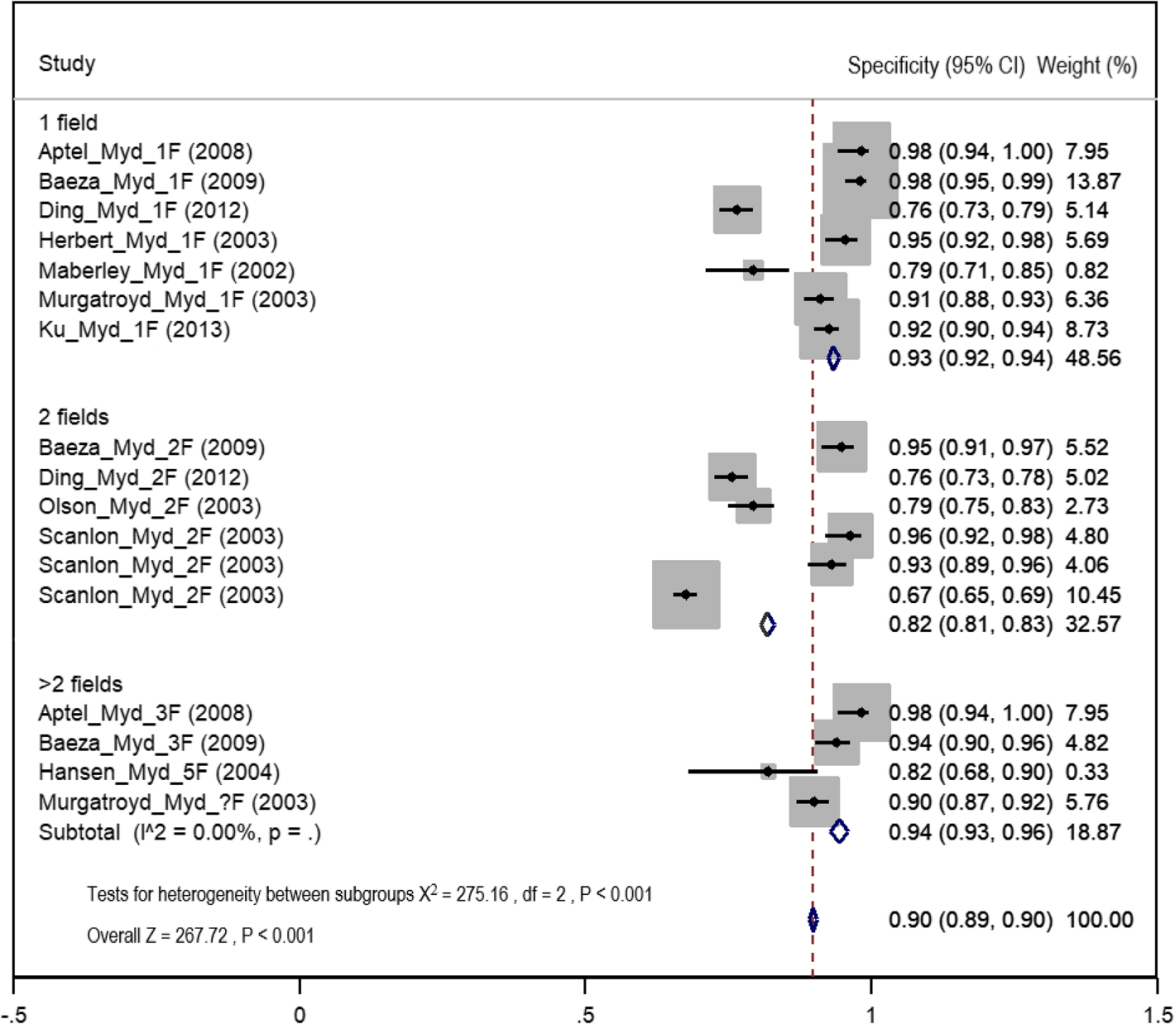
Forest plot of summary estimates of specificity of mydriatic imaging using different field strategies (1: one field, 2: two fields, 3: greater than two fields)

The highest pooled sensitivity of detection of any level of DR using different mydriatic digital imaging field strategies was for the greater than two field strategy (92%, 95% CI 90–94%). The sensitivity of the one-field strategy was 80% (95% CI 77–82%), and it was 85% (95% CI 84–87%) for the two-field strategy (Fig. 6, Table 3). The mean proportion of ungradable images for the mydriatic method was 6.2% (SE± 2.2, 95% CI 1.7–10.8%). The summary estimation of specificity in detection of any level of DR using mydriatic digital imaging was highest in the greater than two field strategy at 94% (95% CI 93–96%) followed by the one field, 93% (95% CI 92–94%) and then two field 82% (95% CI 81–83%) (Fig. 7, Table 3).

### Hierarchical summary receiver operating characteristics (HSROC) curve interpretation

**Fig. 8:**
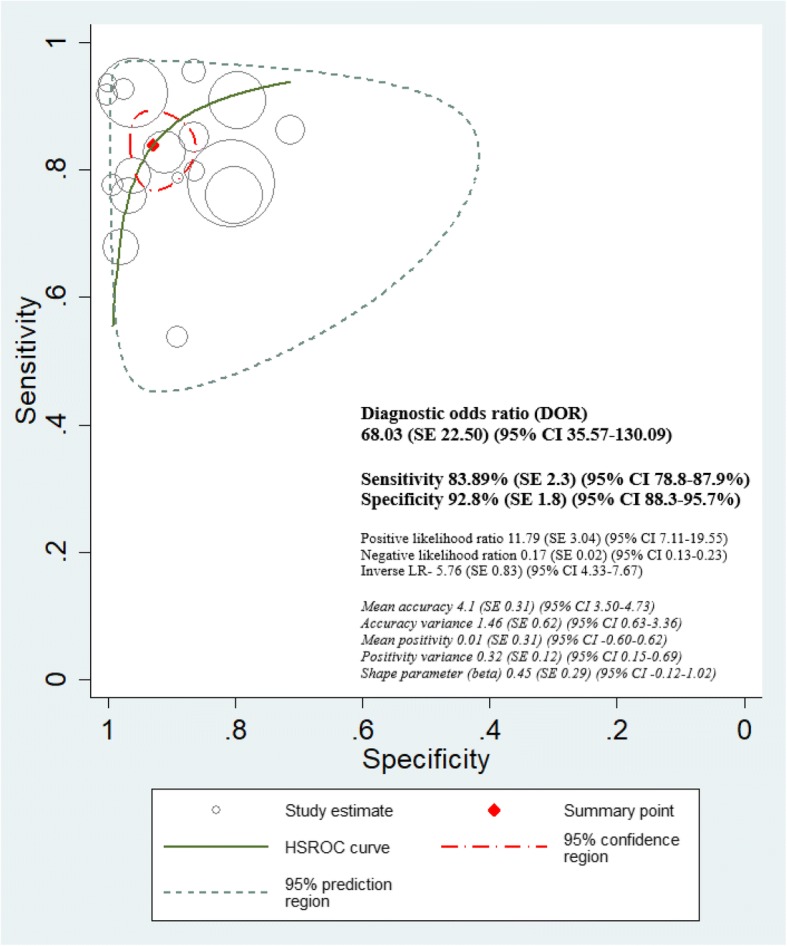
HSROC curve in non-mydriatic imaging strategies

**Fig. 9:**
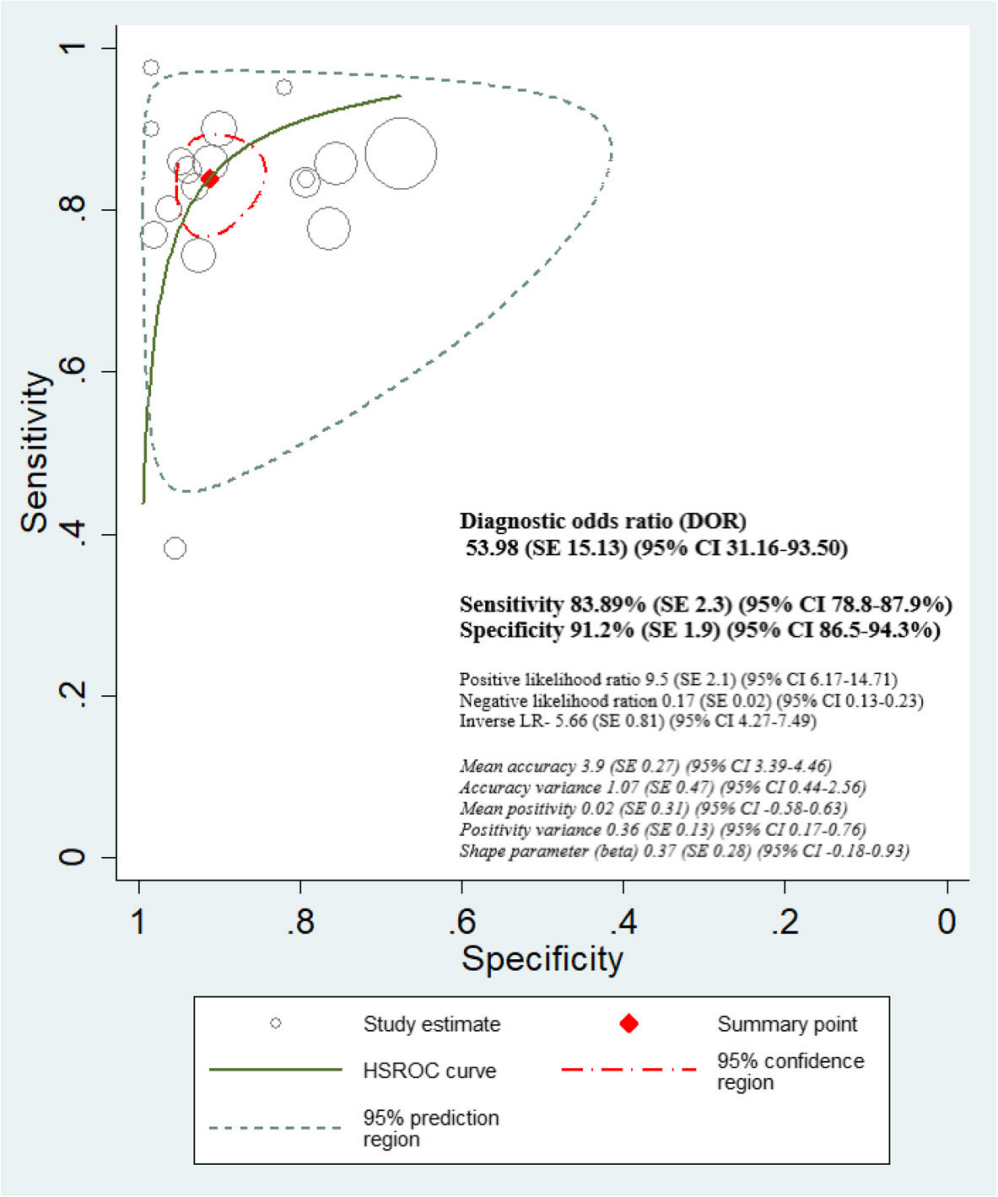
HSROC curve in mydriatic imaging strategies

**Fig. 10:**
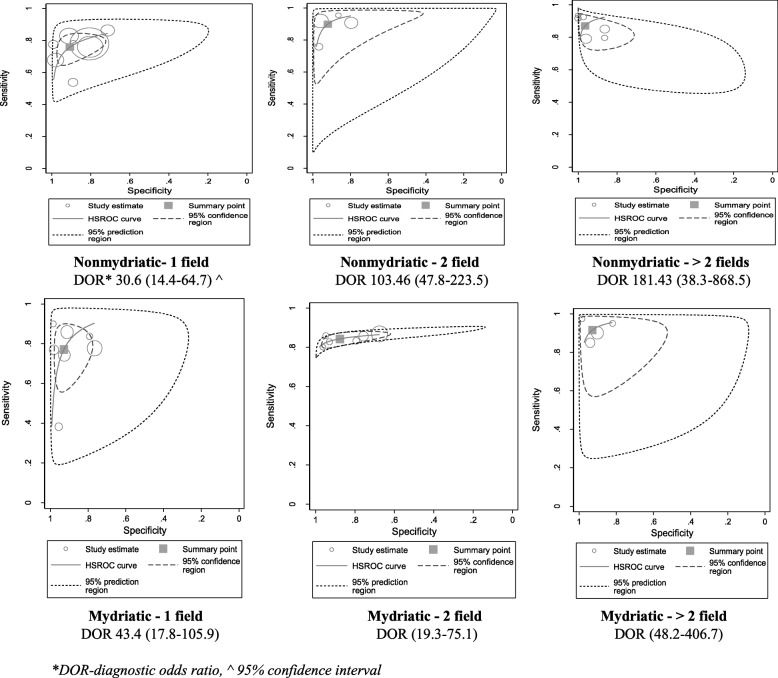
HSROC curves by field strategy and pupil status

Both non-mydriatic and mydriatic strategies showed very high discriminative power in ruling out the presence or absence of any level of DR with the diagnostic odds ratio (DOR) of non-mydriatic strategies being 68.03 (95% CI 35.5–130.0) and positive likelihood ratio of 11.79 (SE 3.04, 95% CI 7.1–19.5) (Fig. 8). Similarly, mydriatic DOR was 53.98 (95% CI 31.1–93.5) and the positive likelihood ratio was 9.5 (SE 2.1, 95% CI 6.1–14.7) (Fig. 9). After adjusting for ungradable images, we observed that the pooled sensitivity of detection of any level of DR was the same for non-mydriatic and mydriatic strategies: 86% (95% CI 85–87%) for both. The specificity of detection of any level of DR was higher using both non-mydriatic and mydriatic greater than two field strategies (94%, 95% CI 93–96%) and in two-field non-mydriatic strategy (94%, 95% CI 93–95%). The highest DOR was obtained for the greater than two field strategy (non-mydriatic DOR 182.4 (SE 145.2, 95% CI 38.3–868.5), mydriatic DOR 140 (SE 76.1, 95% CI 48.2–406.7)). Therefore, we have to consider the number of fields in a DRS strategy (Fig. 10 and Additional file 5).

Summary estimates were derived by the reference test, to assess the variability in DTA according to the reference standard. The pooled sensitivity of detection of any level of DR was higher in non-mydriatic imaging using seven-field ETDRS images as the reference than direct/indirect ophthalmoscopy: (87%, 95% CI 85–89% vs 86%, 95% CI 85–88% in mydriatic). There was no significant difference when compared with mydriatic bio-microscopic ophthalmoscopy as the reference standard (non-mydriatic 86%, 95% CI 85–88% vs mydriatic 86%, 95% CI 85–87%). Pooled estimates of specificity were high in both non-mydriatic (96%, 95% CI 95–97%) and mydriatic (96%, 95% CI 95–97%) imaging using seven-field ETDRS images as the reference standard compared to mydriatic bio-microscopy (non-mydriatic 91%, 95% CI 91–92 vs mydriatic 87%, 95% CI 86–88%) (Table 3 and forest plots available in Additional file 6).

In the analysis of DTA by setting, the highest estimates were shown in secondary/tertiary settings using non-mydriatic imaging (sensitivity 90%, 95% CI 88–91; specificity 95%, 95% CI 94–96%) compared to mydriatic imaging (sensitivity 87%, 95% CI 86–89%; specificity 89%, 95% CI 88–90%) (Table 4). However, in non-mydriatic methods, there was one study from HIC with a larger sample size, which may have attributed for a skewed result (40).

**Table 4:** Summary estimates of diagnostic test accuracy by field strategy, by index test grader, by pupil status and by setting

Imaging strategy		Index grader	Non-mydriatic				Mydriatic			
			Sensitivity		Specificity		Sensitivity		Specificity	
			Estimate (95% CI)	Number of studies	Estimate (95% CI)	Number of studies	Estimate (95% CI)	Number of studies	Estimate (95% CI)	Number of studies
Overall estimates			86% (85–87%)	18	93% (92–93%)	18	86% (85–87%)	17	90% (89–90%)	17
By index test grader		Ophthalmologist	82% (80–84%)	7	94% (94–95%)	7	87% (85–89%)	9	93% (92–94%)	9
		Retinologist	90% (89–92%)	7	94% (93–95%)	7	69% (62–75%)	2	93% (91–96%)	2
		Retinal reader	89% (86–93%)	3	91% (88–94%)	3	86% (84–88%)	4	92% (91–94%)	4
		SpR registrar	78% (75–81%)	1	81% (79–82%)	1	86% (84–89%)	2	70% (68–72%)	2
By field strategy	1F	Ophthalmologist	73% (69–77%)	3	96% (94–97%)	3	78% (75–81%)	4	94% (92–95%)	4
		Retinologist	79% (75–84%)	3	81% (76–86%)	3	69% (62–75%)	2	93% (91–96%)	2
		Retinal reader	83% (77–87%)	1	91% (88–94%)	1	86% (81–89%)	1	91% (88–93%)	1
		SpR registrar	78% (75–81%)	1	81% (79–82%)	1	No data	–	No data	–
	2F	Ophthalmologist	87% (84–90%)	2	90% (88–92%)		86% (83–89%)	2	86% (84–88%)	2
		Retinologist	93% (91–95%)	2	96% (94–97%)	2	No data		No data	
		Retinal reader	No data	–	No data	–	82% (78–85%)	2	95% (92–97%)	2
		SpR registrar	No data	–	No data	–	86% (84–89%)	2	70% (68–72%)	2
	> 2F	Ophthalmologist	84% (79–88%)	2	97% (95–98%)	2	93% (91–96%)	3	96% (94–98%)	2
		Retinologist	82% (75–90%)	2	86% (8–90%)	2	No data	–	No data	–
		Retinal reader	93% (89–96%)	2	No data	–	90% (86–93%)	1	90% (87–92%)	1
		SpR registrar	No data	–	No data	–	No data	–	No data	–
By setting		Primary	85% (83–86%)	8	92% (91–92%)	8	82% (80–84%)	6	91% (90–92%)	6
		Other (secondary or tertiary)	90% (88–91%)	10	95% (94–96%)	10	87% (86–89%)	11	89% (88–90%)	11

Regarding the personnel involved in index test grading, for ‘any level’ of DR, the highest pooled sensitivity and specificity using non-mydriatic imaging was reported by retinologists: sensitivity 90% (95% CI 89–92%) and specificity 94% (95% CI 93–95%). The highest DTA estimates in mydriatic imaging were reported by ophthalmologists (sensitivity 87%, 95% CI 85–89%; specificity 93%, 95% CI 92–94%) (Table 4 and forest plots available in Additional file 7).

### Secondary output

In the sub-analysis of those studies that captured images of the same participant before and after pupil dilatation, mydriasis (one field, two fields, three fields and five field: six studies, ten estimates) showed a high level of sensitivity: mydriatic 88% (95% CI 86–89) and non-mydriatic 82% (95% CI 80–84%). However, a higher level of specificity was shown in non-mydriatic methods in detecting any level of DR: non-mydriatic 92% (95% CI 91–93%) and mydriatic 89% (95% CI 88–90%). Forest plots of these estimates are available in Additional file 8. Four studies used non-ophthalmologist personnel as primary graders in the index test. The pooled sensitivity and specificity of detecting any level of DR (either non-mydriatic or mydriatic) were 74% (95% CI 71–77%) and 85% (95% CI 83–87%) respectively (27, 28, 46, 48).

## Discussion

Overall, both mydriatic and non-mydriatic digital imaging methods generate a satisfactory level of sensitivity, i.e. 86% (95% CI 85–87%) in usual clinical settings, once ungradable images are excluded from analysis. This sensitivity level is above the DRS recommendation of established national programmes (sensitivity > 80%) [50]. Neither strategies achieved the recommended level of 95% specificity for any level of DR: non-mydriatic 95% CI (92–93%) and mydriatic 95% CI (89–90%). In addition, mydriatic greater than two field strategy showed the highest level of sensitivity (92%, 95% CI 90–94) and specificity (94%, 95% CI 93–96%), a finding to be considered when setting up a screening strategy.

The optimum level of referable DR will depend on the accuracy of the screening strategy chosen and the resources available in the specific screening setting in order to strike a balance between screening PwDM at non-ophthalmic settings safely, but without overloading the eye clinics for further assessments. Annual DRS, followed by timely treatment of those confirmed to have STDR is the recommended screening pathway [51]. The current method of DRS in most LMICs is an opportunistic screening using mydriatic bio-microscopic ophthalmoscopy by an ophthalmologist [18]. This is not an efficient way of screening for DR considering the limitations in human resources and access barriers. In contrast, DRS using digital imaging requires specific training and skills, but these can be obtained by non-medical personnel, and as such the pool of potential workforce is much larger than for trained ophthalmologists.

In this meta-analysis, we aimed to show the effect of pupil status on DTA for any DR. For those images sets with gradable images, the pooled sensitivity of non-mydriatic strategies was the same as that of the mydriatic strategies. However, only six studies (6/21) used the same participants before and after pupil dilatation [25, 35, 40, 42, 44, 47]. The non-mydriatic method results were primarily dominated by one larger study (sample size *n* = 1549) conducted in a HIC [40] and another study used wide field (Optomap® 180–200° field view) imaging [26]. Therefore, the outcome of this review should be applied to LMICs cautiously. A similar result was reported in a meta-analysis by Bragge et al. although heterogeneity among those studies was high due to pooling of different examination techniques in one estimation [17]. In the current meta-analysis, heterogeneity was minimised by including studies which used digital retinal imaging only in the index test.

A DRS method which is suited to the health system is a key factor in the success of a programme. Non-mydriatic imaging can be used in settings where there are fewer ophthalmic personnel and avoiding pupil dilatation reduces screening time and causes less perceived inconvenience to PwDM. A concern, however, is variability in image quality, particularly in populations with a high prevalence of cataract and corneal opacities [14, 52]. The Scottish National Health Services DRSP now uses non-mydriatic imaging systems, with minimal need for pupil dilatation in screened patients [53]. This is an evidence-based pragmatic approach with greater convenience for PwDM and lower cost to service providers [54, 55]. However, implementation of non-mydriatic test in DRS will depend on population characteristics such as the prevalence of cataract.

Selection of suitable personnel for DRS and grading depends on workforce capacity and availability. DRS by ophthalmologists is not an efficient way of screening for any setting [55]. DM-related blindness is still on the rise everywhere in the world and is a public health concern in LMIC settings as well [18]. These countries will have to rapidly adopt clinically safe and cost-effective strategies to address this issue, using the limited resources available and establish such a programme quickly [56]. In this analysis, retinal image graders could achieve the recommended level of 80% sensitivity and specificity closer to 95% in both mydriatic and non-mydriatic strategies. Therefore, it is justifiable to train non-ophthalmic personnel in DR grading, just as it was done in the UK national programme.

DR screening’s success depends on the gradability of images, as such most of the studies included only gradable images. High population coverage with good quality gradable images is an important pragmatic consideration to achieve high DTA and high acceptability of a DRSP. Therefore, interpretation of the results shown in this study requires judgement of the context and objectives of a specific DRSP. PwDM with ungradable images are a special category of people whose fundus is not visible due to some other ocular pathology like dense lenticular opacities. These people therefore not only need the management that test negatives receive in terms of management of diabetic retinopathy but will also need additional management of ocular pathology which is obliterating the fundus image. Therefore, this meta-analysis highlights the concerns as to how to manage data on ungradable images, as studies differ in their approach of dealing with such a concern. Most authors (13 studies) had excluded ungradable images from their analysis while others included them as having screened positive (six studies). In addition, reporting of ungradable by study authors was heterogeneous, which imply requirement of standardised reporting of ungradable images in DRS.

The mean proportions of ungradable images in non-mydriatic and mydriatic imaging were 17.8% (95% CI 10.8–24.8%) and 6.1% (95% CI 3.7–8.4%) respectively. The decisions made by each study authors may have introduced reporting bias in their measures of DTA. Considering ungradable images as test positives may have led to inflated estimates of DTA in some studies [25, 26, 40, 42–44]. The mean proportions of ungradable images included by study authors as test positives in non-mydriatic and mydriatic imaging were 12.5% (95% CI 9.0–16.1%) and 2.5% (95% CI 1.0–3.9%) respectively. Therefore, we adjusted DTA to take account of ungradable images by excluding those to reduce heterogeneity. This was possible for four of the six studies in which ungradable images were included as screening positive [25, 26, 40, 43], but two did not report adequate data to allow for this [42, 44]. As an example, we made adjustment (calculated sensitivity 42/49 = 85.7%, specificity 227/262 = 86.6%) for the inflated DTA (reported sensitivity 98%, specificity 100%) in the study of Ahmed et al. using the 2 × 2 table data reported by study authors [29]. In another two studies, it was not clear how ungradable images had been managed [28, 38]. The proportions of ungradable images and DTA after adjustments in each strategy are available in Additional file 9.

### Limitations

The definition of ungradable images was not uniform in the studies included in the current review We minimised the heterogeneity by excluding the ungradable images and by sub-group analysis.

The studies which used non-mydriatic imaging techniques were more recent, being conducted after rapid advancements in technology for such imaging technology leading to better quality images using non-mydriatic systems without pupil dilatation as well and a major confounder in the meta-analysis.

The results of the different strategies described in this review are to be considered fully if a comprehensive DRSP facilitating greater screening coverage with improved accessibility and good quality imaging is to be set up. However, due to lack of relevant good quality data, sub-analysis by countries’ income setting was not possible to perform due to absence of studies from LMICs.

We excluded three articles which were not in English due to practical barriers in translations and assessment of methodological quality.

The DTA of detection of maculopathy had not been considered. The maculopathy is also an important aspect in DRS, and it may have to be considered in a separate review.

## Conclusions

Diagnostic test accuracy for the detection of any level of DR showed that DRS using two fields delivered at non-primary care settings is a feasible approach. Dilatation of the pupils did not improve the detection of any level of DR for those with gradable images, but such a wide range of ungradable were presented in these studies that this aspect must be taken into account when setting up DRSP. There was no adequate evidence in primary studies to comment on DTA of non-ophthalmological human resources on DRS, so this aspect requires further research. Good quality digital imaging has the potential for real-time interpretation of retinal images, which together with counselling for risk factors may improve the acceptability of DRS and uptake of referral for ophthalmic assessment if conducted in a culturally acceptable way.

### Recommendations

Diagnostic test accuracies of the newer non-mydriatic imaging systems should be further explored in different environments and using a different skill-mix of graders, especially in LMICs.

Studies should focus on the accuracy of non-ophthalmic graders and non-ophthalmic settings to explore the potential of initiating DRSP especially in low-income settings. This will reduce the number of referrals to eye departments, many of which are already over-burdened with cataract and other eye conditions, particularly in LMIC where resources are limited.

The reporting definitions of technical failures or ungradability of the images should be standardised using a reporting guideline.

A systematic review and meta-analysis of DTA of different levels of DR and maculopathy can be recommended in future research.

## Additional files

Additional file 1: PRISMA check list. (DOCX 21 kb)

Additional file 2: Details of the excluded studies. (DOCX 49 kb)

Additional file 3: Participant characteristics of the included articles. (DOCX 28 kb)

Additional file 4: DTA of different strategies and ungradable image proportions as reported by study authors. (DOCX 29 kb)

Additional file 5: DTA parameters by pupil status and field strategy using HSROC curves. (DOCX 20 kb)

Additional file 6: Forest plots of DTA variation by type of reference standard and by the level of service delivery (by clinic settings). (DOCX 536 kb)

Additional file 7: Forest plots of DTA by different index test human resources. (DOCX 304 kb)

Additional file 8: Forest plots of sub-analyses—DTA using same participant undergoing imaging before and after pupil dilatation. (DOCX 162 kb)

Additional file 9: DTA following adjustments in relevant to exclusion of ungradable proportions in the current review. (DOCX 27 kb)

## Abbreviations

DM: Diabetes mellitus
DR: Diabetic retinopathy
DTA: Diagnostic test accuracy
ETDRS: Early treatment diabetic retinopathy study
HIC: High-income country
LIC: Low-income country
LMIC: Low- and middle-income country
NPDR: Non-proliferative diabetic retinopathy
PwDM: People with diabetes mellitus
QUADAS: Quality assessment for diagnostic accuracy studies
STDR: Sight-threatening diabetic retinopathy

## References

[1] Whiting DR, Guariguata L, Weil C, Shaw J (2011). Global estimates of the prevalence of diabetes for 2011 and 2030. Diabetes Res Clin Pract.

[2] Diabetes - International Diabetes Federation. 2017. http://www.idf.org/idf-diabetes-atlas-seventh-edition. Accessed 10 Jan 2018. 10.1111/1753-0407.12644 29345068

[3] Shaw JE, Sicree RA, Zimmet PZ (2010). Global estimates of the prevalence of diabetes for 2010 and 2030. Diabetes Res Clin Pr.

[4] Guariguata L, Whiting DR, Hambleton I, Beagley J (2013). Global estimates of diabetes prevalence for 2013 and projections for 2035. Diabetes Res Clin Pract.

[5] Klein Ronald, Klein Barbara E.K. (2013). The Epidemiology of Diabetic Retinopathy. Retina.

[6] Yau JWY, Rogers SL, Kawasaki R, Lamoureux EL, Kowalski JW, Bek T (2012). Global prevalence and major risk factors of diabetic retinopathy. Diabetes Care.

[7] Zhang X, Norris SL, Saadine J, Chowdhury FM, Horsley T, Kanjilal S (2007). Effectiveness of interventions to promote screening for diabetic retinopathy. Am J Prev Med.

[8] Klein BE (2007). Overview of epidemiologic studies of diabetic retinopathy. Ophthalmic Epidemiol.

[9] The Diabetes Control and Complications Trial Research Group (1993). The effect of intensive treatment of diabetes on the development and progression of long term complications in insulin-dependent diabetes mellitus. N Engl J Med.

[10] Klein R, Knudtson MD, Lee KE, Gangnon R, Klein BEK (2009). The Wisconsin epidemiologic study of diabetic retinopathy xxii. The twenty-five-year progression of retinopathy in persons with type 1 diabetes. NIH public access. Diabetes.

[11] Kohner EM, Aldington SJ, Stratton IM, Manley SE, Holman RR, Mathews DR, Turner RC (1998). United Kingdom Prospective Diabetes Study, 30: diabetic retinopathy at diagnosis of non-insulin-dependent diabetes mellitus and associated risk factors. Arch Ophthalmol.

[12] The Diabetic Retinopathy Study Research Group (1976). Photocoagulation treatment of proliferative diabetic retinopathy. Ophthalmology.

[13] Scanlon PH (2017). The English National Screening Programme for diabetic retinopathy 2003–2016. Acta Diabetol.

[14] Banaee T, Ansari-Astaneh MR, Pourreza H, Faal Hosseini F, Vatanparast M, Shoeibi N (2017). Utility of 1% tropicamide in improving the quality of images for tele-screening of diabetic retinopathy in patients with dark irides. Ophthalmic Epidemiol.

[15] Hutchinson A, McIntosh A, Peters J, Keeffe JE, Khunti K, Baker R (2000). Effectiveness of screening and monitoring tests for diabetic retinopathy - a systematic review. Diabet Med.

[16] Shi L, Wu H, Dong J, Jiang K, Lu X, Shi J (2015). Telemedicine for detecting diabetic retinopathy: a systematic review and meta-analysis. Br J Ophthalmol.

[17] Bragge P, Gruen RL, Chau M, Forbes A, Taylor HR (2011). Screening for presence or absence of diabetic retinopathy. Arch Ophthalmol.

[18] Lin Stephanie, Ramulu Pradeep, Lamoureux Ecosse L, Sabanayagam Charumathi (2016). Addressing risk factors, screening, and preventative treatment for diabetic retinopathy in developing countries: a review. Clinical & Experimental Ophthalmology.

[19] Hipwell AE, Sturt J, Lindenmeyer A, Stratton I, Gadsby R, Hare PO (2014). Attitudes, access and anguish: a qualitative interview study of staff and patients ’ experiences of diabetic retinopathy screening. BMJ Open.

[20] von Elm E, Altman DG, Egger M, Pocock SJ, Gøtzsche PCVJSI (2007). The Strengthening the Reporting of Observational Studies in Epidemiology (STROBE) statement: guidelines for reporting observational studies. PLoS Med.

[21] Macaskill P, Gatsonis C, Deeks J, Harbord R, Takwoingi Y. Cochrane handbook for systematic reviews of diagnostic test accuracy chapter 10 analysing and presenting results. Cochrane DTA Handb. 2010:1–61 http://methods.cochrane.org/sites/methods.cochrane.org.sdt/files/public/uploads/Chapter%2010%20-%20Version%201.0.pdf. Accessed 30 Sept 2015.

[22] Whiting PF, Rutjes AWS, Westwood ME, Mallet S, Deeks JJ, Reitsma JB (2011). Research and reporting methods accuracy studies. Ann Intern Med.

[23] Herbert HM, Jordan K, Flanagan DW (2003). Is screening with digital imaging using one retinal view adequate?. Eye (Lond).

[24] Olson JA, Strachan FM, Hipwell JH, Goatman KA, McHardy KC, Forrester JV (2003). A comparative evaluation of digital imaging, retinal photography and optometrist examination in screening for diabetic retinopathy. Diabet Med.

[25] Hansen AB, Sander B, Larsen M, Kleener J, Borch-Johnsen K, Klein R (2004). Screening for diabetic retinopathy using a digital non-mydriatic camera compared with standard 35-mm stereo colour transparencies. Acta Ophthalmol Scand.

[26] Neubauer AS, Kernt M, Haritoglou C, Priglinger SG, Kampik A, Ulbig MW (2008). Nonmydriatic screening for diabetic retinopathy by ultra-widefield scanning laser ophthalmoscopy (Optomap). Graefes Arch Clin Exp Ophthalmol.

[27] Henricsson M, Karlsson C, Ekholm L, Kaikkonen P, Sellman A, Steffert E (2000). Colour slides or digital photography in diabetes screening--a comparison. Acta Ophthalmol Scand.

[28] Sundling V, Gulbrandsen P, Straand J (2013). Sensitivity and specificity of Norwegian optometrists’ evaluation of diabetic retinopathy in single-field retinal images - a cross-sectional experimental study. BMC Health Serv Res.

[29] Ahmed J, Ward TP, Bursell SE, Aiello LM, Cavallerano JD, Vigersky RA (2006). The sensitivity and specificity of nonmydriatic digital stereoscopic retinal imaging in detecting diabetic retinopathy. Diabetes Care.

[30] Shiffman R, Jacobsen G, Nussbaun J, Desai U, Carey D, Glasser D (2005). Comparison of a digital retinal imaging system and seven-field stereo colour fundus photography to detect diabetic retinopathy in the primary care environment. Ophthalmic Surg Lasers Imaging.

[31] Boucher MC, Gresset JA, Angioi K, Olivier S (2003). Effectiveness and safety of screening for diabetic retinopathy with two nonmydriatic digital images compared with the seven standard stereoscopic photographic fields. Can J Ophthalmol.

[32] Maberley D, Cruess AF, Barile G, Slakter J (2002). Digital photographic screening for diabetic retinopathy in the James Bay Cree. Ophthalmic Epidemiol.

[33] Perrier M, Boucher MC, Angioi K, Gresset JA, Olivier S (2003). Comparison of two, three and four 45 degrees image fields obtained with the Topcon CRW6 nonmydriatic camera for screening for diabetic retinopathy. Can J Ophthalmol.

[34] Bhargava M, Cheung CYL, Sabanayagam C, Kawasaki R, Harper CA, Lamoureux EL (2012). Accuracy of diabetic retinopathy screening by trained non-physician graders using non-mydriatic fundus camera. Singap Med J.

[35] Murgatroyd H (2004). Effect of mydriasis and different field strategies on digital image screening of diabetic eye disease. Br J Ophthalmol.

[36] Mizrachi Yossi, Knyazer Boris, Guigui Sara, Rosen Shirley, Lifshitz Tova, Belfair Nadav, Klemperer Itamar, Schneck Marina, Levy Jaime (2013). Evaluation of diabetic retinopathy screening using a non-mydriatic retinal digital camera in primary care settings in south Israel. International Ophthalmology.

[37] Ku JY, Landers J, Henderson T, Craig JE (2013). The reliability of single-field fundus photography in screening for diabetic retinopathy: the central Australian ocular health study. Med J Aust.

[38] Phiri R., Keeffe J. E., Harper C. A., Taylor H. R. (2006). Comparative study of the polaroid and digital non-mydriatic cameras in the detection of referrable diabetic retinopathy in Australia. Diabetic Medicine.

[39] Scanlon PH, Malhotra R, Greenwood RH, Aldington SJ, Foy C, Flatman M (2003). Comparison of two reference standards in validating two field mydriatic digital photography as a method of screening for diabetic retinopathy. Br J Ophthalmol.

[40] Scanlon PH, Malhotra R, Thomas G, Foyt C, Kirkpatrick JN, Lewis-Barned N (2003). The effectiveness of screening for diabetic retinopathy by digital imaging photography and technician ophthalmoscopy. Diabet Med.

[41] Tu K, Palmer P, Sen S, Matthew P, Khaleel A (2004). Comparison of optometry vs digital photography screening for diabetic retinopathy in a single district. Eye.

[42] Aptel F, Denis P, Rouberol F, Thivolet C (2008). Screening of diabetic retinopathy: effect of field number and mydriasis on sensitivity and specificity of digital fundus photography. Diabetes Metab.

[43] Massin P, Erginay A, Ben Mehidi A, Vicaut E, Quentel G, Victor Z (2003). Evaluation of a new non-mydriatic digital camera for detection of diabetic retinopathy. Diabet Med.

[44] Baeza M, Orozco-Beltrán D, Gil-Guillen VF, Pedrera V, Ribera MC, Pertusa S (2009). Screening for sight threatening diabetic retinopathy using non-mydriatic retinal camera in a primary care setting: to dilate or not to dilate?. Int J Clin Pract.

[45] Lopez-Bastida J, Cabrera-Lopez F, Serrano-Aguilar P (2007). Sensitivity and specificity of digital retinal imaging for screening diabetic retinopathy. Diabet Med.

[46] Suansilpong A, Rawdaree P (2008). Accuracy of single-field nonmydriatic digital fundus image in screening for diabetic retinopathy. J Med Assoc Thail.

[47] Ding Jiyuan, Zou Yanhong, Liu Ningpu, Jiang Li, Ren Xuetao, Jia Wei, Snellingen Torkel, Chongsuvivatwong Virasakdi, Liu Xipu (2012). Strategies of Digital Fundus Photography for Screening Diabetic Retinopathy in a Diabetic Population in Urban China. Ophthalmic Epidemiology.

[48] Kuo HK, Hsieh HH, Liu RT (2005). Screening for diabetic retinopathy by one-field, non-mydriatic, 45° digital photography is inadequate. Ophthalmologica.

[49] Massin P, Aubert JP, Eschwege E, Erginay A, Bourovitch JC, BenMehidi A (2005). Evaluation of a screening program for diabetic retinopathy in a primary care setting Dodia (Dépistage ophtalmologique du diabète) study. Diabetes Metab.

[50] Mead A, Burnett S, Davey C (2001). Diabetic retinal screening in the UK. Journal of the Royal Society of Medicine.

[51] Taylor-Phillips S, Mistry H, Leslie R, Todkill D, Tsertsvadze A, Connock M (2016). Extending the diabetic retinopathy screening interval beyond 1 year: systematic review. Br J Ophthalmol.

[52] Scanlon P. H., Foy C., Malhotra R., Aldington S. J. (2005). The Influence of Age, Duration of Diabetes, Cataract, and Pupil Size on Image Quality in Digital Photographic Retinal Screening. Diabetes Care.

[53] The Diabetic Retinopathy Screening Implementation Group (2003). Diabetic retinopathy screening services in Scotland: recommendations for implementation.

[54] Guigui S, Lifshitz T, Levy J (2012). Screening for diabetic retinopathy: review of current methods. Hosp Pract.

[55] Swanson M (2005). Retinopathy screening in individuals with type 2 diabetes: who, how, how often, and at what cost--an epidemiologic review. Optometry.

[56] Khandekar R (2012). Screening and public health strategies for diabetic retinopathy in the Eastern Mediterranean Region. Middle East Afr J Ophthalmol.

