# Correction to: Systematic review and meta-analysis of diagnostic accuracy of detection of any level of diabetic retinopathy using digital retinal imaging

**DOI:** 10.1186/s13643-019-1023-7

**Published:** 2019-04-30

**Authors:** Mapa Mudiyanselage Prabhath Nishantha Piyasena, Gudlavalleti Venkata S. Murthy, Jennifer L. Y. Yip, Clare Gilbert, Tunde Peto, Iris Gordon, Suwin Hewage, Sureshkumar Kamalakannan

**Affiliations:** 1 Clinical Research Department, International Centre for Eye Health, London School of Hygiene and Tropical Medicine, Keppel Street, London, WC1E 7HT UK; 2 School of Medicine, Dentistry and Biomedical Sciences, Queen’s University, 97, Lisburn Road, Belfast, BT9 7BL Northern Ireland; 3 Retina Research Unit, National Eye Hospital, Deans Road, Colombo, 01000 Sri Lanka; 4 Indian Institute of Public Health, Plot No 1 Kavuri Hills Madhapur, Hyderabad, 500033 India

**Correction to: Syst Rev**

**https://doi.org/10.1186/s13643-018-0846-y**

Following publication of the original article [1], the authors reported an error in Fig. 4 in the PDF version. Figure 4 is the duplicate image of Fig. 3 and the correct figure is missing. The authors would like to apologize for this error. The correct figure is shown below.

**Fig. 4:**
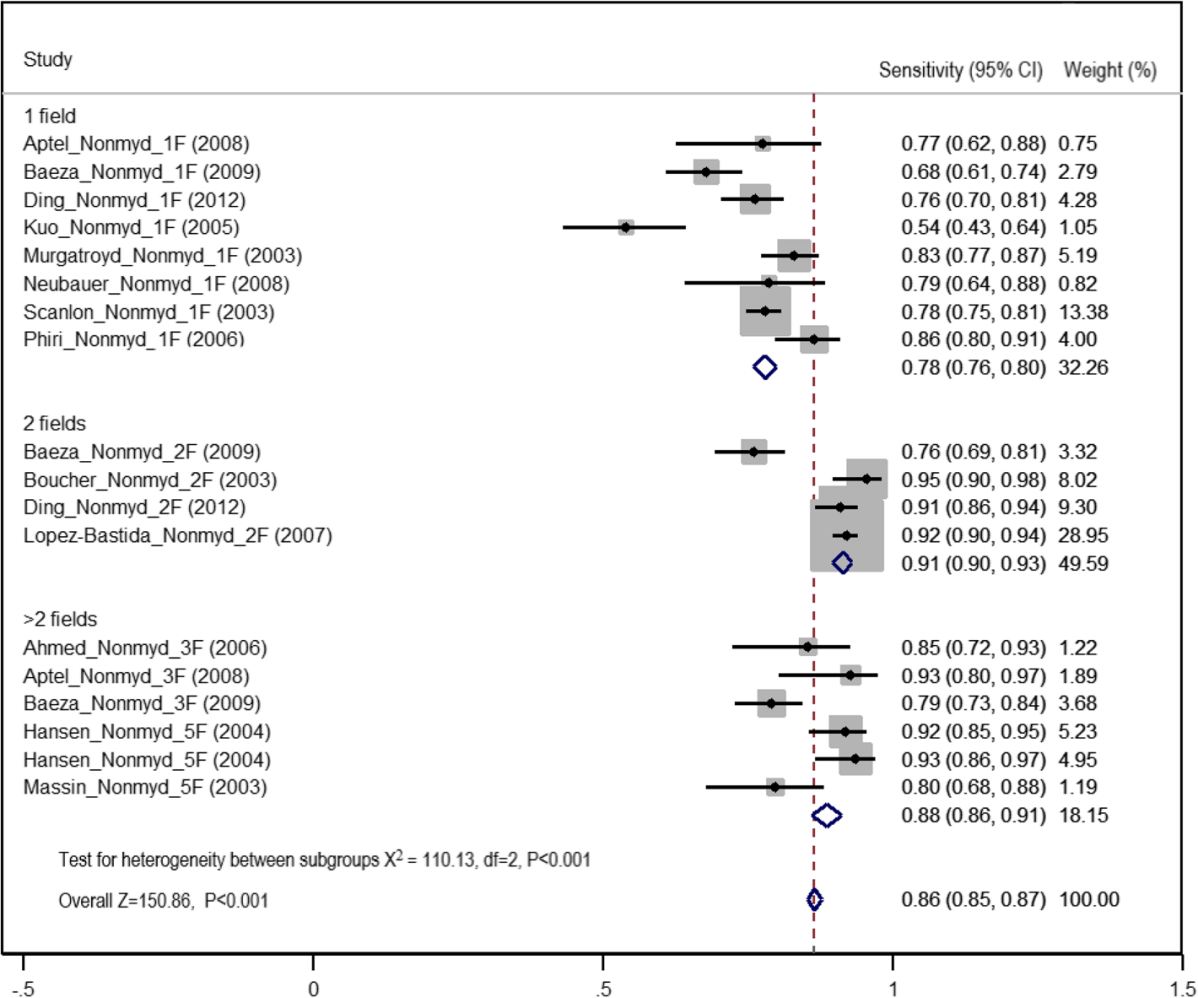
Forest plot of summary estimates of sensitivity of non-mydriatic imaging using different field strategies (1: one field, 2: two fields, 3: greater than two fields)
