# Supplementary material for: Systematic review and meta-analysis of diagnostic accuracy of detection of any level of diabetic retinopathy using digital retinal imaging
Source: Syst Rev. 2018 Nov 7;7:182. doi: 10.1186/s13643-018-0846-y (PMC6222985; doi:10.1186/s13643-018-0846-y)
Supplement: Supplementary file 2 — Details of the excluded studies. (DOCX 49 kb) [file 13643_2018_846_MOESM2_ESM.docx]

**Additional file 2 – Table 1 –** **Study setting, reasons for exclusion and DTA reported in studies excluded from current review -**

| **Study Source** | **Type of Study** | **Setting** | **Screening Strategy** | **Sample Size** | **Reason for Exclusion** | **Index Test Grader** | **Reference Standard** | **DTA Values (Sensitivity)** | **DTA Values (Specificity)** |
| --- | --- | --- | --- | --- | --- | --- | --- | --- | --- |
| 1.Al Sabti, K. et al 2003 (Kuwait) | Cross sectional | Retina clinic | Digital fundus photography (Mydriatic, 30^0^ and 60^0^) | 51 | Not a study assessing sensitivity/specificity | Examiners | Dilated slit lamp examination by retina specialist | Kappa = 0.93 | Not mentioned |
| 2.Andonegui, J. et al 2008 (Spain) | Interventional | Primary Care | Digital fundus photography | 4 primary care physicians | Full article not in English | Primary care physicians | Same images graded by ophthalmologists | Kappa = 0.8 to 0.95 | Not mentioned |
| 3.Andonegui, J. et al 2012 (Spain) | Audit | Primary Care | Digital fundus photography (Mydriatic and Non-mydriatic) | 2750 | An audit. Does not involve a proper reference standard | General practitioners | Same images graded by ophthalmologists | False positive = 55% | False negative = 7% |
| 4.Anonymous 1986 | Not relevant | Not relevant | Not relevant | Not relevant | A letter to the editor | Not relevant | Not relevant | Not relevant | Not relevant |
| 5.Anonymous 1988 | Not relevant | Not relevant | Not relevant |  | No such article available | Not relevant | Not relevant | Not relevant | Not relevant |
| 6.Awan, A. M. et al 1974 (Kenya) | Cross sectional | National Hospital | Colour fundus photography (Mydriatic, 5F) | 115 | Not a study on DRS DTA | Not mentioned | Not mentioned | Not mentioned | Not mentioned |
| 7.Backlund, L. B. et al 1998 (Sweden) | Cross sectional | Primary Care | Film fundus photography (Mydriatic and non-mydriatic, 4F, 45^0^) | 5490 | Not a study on DRS DTA | Registered ophthalmic nurses and a GP | Not relevant | Not relevant | Not relevant |
| 8.Baeza Diaz, M. et al 2004 (Spain) | Cross sectional | Primary Care | Digital fundus photography | 188 | Full article not in English | Not mentioned | Not mentioned | >75% | >95% |
| 9.Barrie, T. et al 1986 | Not relevant | Not relevant | Not relevant | Not relevant | A letter to the editor | Not relevant | Not relevant | Not relevant | Not relevant |
| 10.Benbassat, J. et al 2009 | Literature review | Not relevant | Not relevant | Not relevant | A literature review | Not relevant | Not relevant | Not relevant | Not relevant |
| 11.Bragge, P. et al 2011 | Meta-analysis | Not relevant | Not relevant |  | A meta-analysis | Not relevant | Not relevant | Not relevant | Not relevant |
| 12.Burns-Cox, C. J. et al 1985 (UK) | Cross sectional | GP, clinic and in-ward | Ophthalmic optician examination | 844 | Does not involve digital retinal imaging | Ophthalmic opticians | Ophthalmologist examination or retinal photography | Not mentioned | Not mentioned |
| 13.Buxton, M. J. et al 1991 (UK) | Cross sectional | Medical centres | Polaroid fundus photography (Non-mydriatic, 45^0^) | 3318 | Does not involve digital retinal imaging | Ophthalmologists | ophthalmoscopic examination by ophthalmological clinical assistant | 35% - 67% | 95% - 98% |
| 14.Bursell, S.E. 2000 (USA) | Cross sectional | Eye care centre | Digital fundus photography (Non-mydriatic, 45^0^, 3F, stereoscopic) | 54 | Digital video imaging. Not relevant to the review question | Two independent readers | 7SF ETDRS | 59% | 80% |
| 15.Carmichael, T. R. et al 2005 (South Africa) | Cross sectional | Diabetic clinic | Film fundus photography (Mydriatic, 60^0^, 1F) | 1595 | Does not involve digital retinal imaging | Endocrinologists | Photographs graded by ophthalmologists | 83% | 99% |
| 16.Cavallerano, J. D. et al 2005 (USA) | Prospective cohort | Eye care centre | Digital fundus photography (Non-mydriatic, 45^0^, 3F, stereoscopic) | 52 | Not a study on DRS DTA | Certified readers | Dilated retinal examination by retinal  Specialist and 7SF ETDRS | Not mentioned | Not mentioned |
| 17.Chalam, K. V. et al 2009 (USA) | Not relevant | Not relevant | Not relevant | Not relevant | A short communication on a new retinal imaging technique | Not relevant | Not relevant | Not relevant | Not relevant |
| 18.Chantelau, E. et al 1989 (Germany) | Cross sectional | Not mentioned | Polaroid fundus photography (Non-mydriatic, 45^0^) | 473 | Does not involve digital retinal imaging | Not mentioned | Not mentioned | 82% | 100% |
| 19.Christopher, M. et al 2012 (USA) | Pilot study | Tertiary care | Images graded using a tablet computer | 1200 | Not a study on DRS. Uses images from a database. | Retinal specialists | Same images graded using a desktop PC | 84.8% | 98.7% |
| 20.Chun, D. W. et al 2007 (USA) | Cross sectional | Primary care | Digital fundus photography (Non-mydriatic, 45^0^, 1F) | 137 | Does not involve onsite grading of images | Ophthalmologist | Dilated retinal examination by retina specialist | 60% | 100% |
| 21.Clements, C. et al 2002 | Not relevant | Not relevant | Not relevant | Not relevant | A letter to the editor | Not relevant | Not relevant | Not relevant | Not relevant |
| 22.de Los Terreros, A. S. et al 2010 (Spain) | Cross sectional | Tertiary care | Digital fundus photography (Non-mydriatic and mydriatic, 30^0^ and 45^0^, 1F, stereoscopic) | 53 | Not a study on DRS DTA. A study on diabetic macular oedema | Endocrinologists | Same images graded by a second endocrinologist | Not relevant | Not relevant |
| 23.de Sonnaville, J. J. et al 1996 (Netherlands) | Cross sectional | Primary care | Film fundus photography (Mydriatic, 60^0^, 2F, black and white) | 323 | Does not involve digital retinal imaging | Not mentioned | Dilated fundoscopy | 97% | 97% |
| 24.Deb-Joardar, N. et al 2005 (France) | Cross sectional | Diabetic care | Digital fundus photography (Non-mydriatic and mydriatic, 45^0^, 5F) | 150 | Not a study on DTA | Endocrinologists | Mydriatic retinal images graded by consensus of ophthalmologists | Not mentioned | Not mentioned |
| 25.Deb-Joardar, N. et al 2007 (France) | Cross sectional | Diabetic care | Digital fundus photography (Mydriatic, 45^0^, 3F) | 1157 | Not a study on DTA | Ophthalmologists | Not relevant | Not relevant | Not relevant |
| 26.Diamond, J. P. et al 1998 (Australia) | Cross sectional | Primary care | Polaroid fundus photography (Non-mydriatic and mydriatic, 45^0^) | 164 | Does not involve digital retinal imaging | Ophthalmologists | Dilated Indirect fundoscopy by ophthalmologist | Kappa = 0.41 | Not mentioned |
| 27.Emanuele, N. et al 2009 (USA) | Cross sectional | Diabetic care | Dilated fundus examination | 340 | Does not involve digital retinal imaging | Ophthalmologists and optometrists | 7SF ETDRS | 51% | 91% |
| 28.Evans, P. M. et al 1997 (UK) | Cross sectional | Primary care | Polaroid fundus photography (mydriatic) | 1010 | Does not involve digital retinal imaging | Ophthalmologist | Ophthalmoscopy by Ophthalmologist | Not mentioned | Not mentioned |
| 29.Farley, TF et al 2008 (USA) | Cross sectional | Community health centre | Polaroid imaging (Mydriatic, 45, 1F) | 1040 | Does not involve digital retinal imaging | Family physicians | Same images graded by a retina specialist | 85% | 94% |
| 30.Feman, S. S. et al 1995 (USA) | Cross sectional | Medical centres | 7SF ETDRS | 2329 | Does not involve digital retinal imaging | Trained non-ophthalmologist research personnel | Same images graded by a different grader or same grader at a later time | Not mentioned | Not mentioned |
| 31.Forrest, R. D. et al 1987 (UK) | Cross sectional | Primary care | Ophthalmoscopy by a trained nurse and diabetologist | 282 | Does not involve digital retinal imaging | Independent assessor at the Retinal Photography Unit | Film fundus photography (Mydriatic, 5F) | Nurse -50.0%,  Doctor - 51.3% | Nurse -99.2%, Doctor – 98.7% |
| 32.George, L. D. et al 1998 (UK) | Cross sectional | Diabetic and ophthalmology care | Digital fundus photography (Mydriatic, 45^0^, 2F) | 40 | Does not involve a proper reference standard. Not a study assessing sensitivity/specificity | Research physician | Film fundus photography (Mydriatic, 45^0^, 2F) graded by a research physician | Kappa = 0.92 | Not mentioned |
| 33.Germain, N. et al 2011 (France) | Cross sectional | Diabetic care | Digital fundus photography (Mydriatic, 45^0^, 3F) | 500 | Does not involve a proper reference standard | Endocrinologists and ophthalmology residents | Same images graded by retina specialists | Endocrinologists -89.7%, ophthalmology residents 96.4% | Endocrinologists - NPV = 91.9, ophthalmology residents NPV = 96.7 |
| 34.Gonzalez, M. E. et al 1995 (Mexico) | Cross sectional | Diabetic care | 7SF ETDRS | 15 | Does not involve digital retinal imaging | Retinal specialists | Same images graded by certified graders | Kappa = 0.53 | Not mentioned |
| 35.Guigui, S. et al 2012 | Literature review | Not relevant | Not relevant | Not relevant | A literature review | Not relevant | Not relevant | Not relevant | Not relevant |
| 36.Harding, S. P. et al 1995 (UK) | Cross sectional | Primary care | Film fundus photography (Mydriatic, 45^0^, 3F) | 395 | Does not involve digital retinal imaging | Ophthalmic clinical assistant | Dilated slit lamp examination by a consultant specialist in medical retinal disease | 89% | 86% |
| 37.Harper, C. A. et al 1998 (Australia) | Cross sectional | Community based | Polaroid fundus photography (Non-mydriatic, 45^0^) | 1177 | Does not involve digital retinal imaging and not a study on DTA | Ophthalmologist | Not relevant | Not relevant | Not relevant |
| 38.Healy, R. et al 2014 (UK) | Cross sectional | DR screening program | Digital fundus photography (Mydriatic, 2F) | 1501 | Not a proper study on DTA as only screening positives were considered in analysis | Nonmedical  graders | Dilated slit lamp examination by ophthalmologist | Not relevant | Not relevant |
| 39.Higgs, E. R. et al 1991 (UK) | Cross sectional | Community based | Film fundus photography (Non-mydriatic) | 405 | Does not involve digital retinal imaging and not a study on DTA | Ophthalmologist | Not relevant | Not mentioned | Not mentioned |
| 40.Jackson, C. L. et al 2002 (Australia) | Interventional | General Practices | Fundal examination | 17 | Does not involve digital retinal imaging | General practitioners | Clinical assessment by ophthalmologists | Post-test – all GPs achieved 50-100% sensitivity | Post-test – 77% of GPs achieved 50-100% specificity |
| 41.Jacob, J. et al 1995 (UK) | Cross sectional | Primary care | Direct and indirect ophthalmoscopy and Polaroid fundus photography (Mydriatic and non-mydriatic, 45^0^) | 1050 | Does not involve digital retinal imaging | Trained non- medically qualified technician | Same images graded by ophthalmologist | Not mentioned | Not mentioned |
| 42.Joannou, J. et al 1996 (South Africa) | Cross sectional | Diabetic care | Film fundus photography (Mydriatic, 60^0^, 1F) | 663 | Does not involve digital retinal imaging | Diabetic clinic doctors | Dilated clinical assessment by an ophthalmologist | 93% | 89% |
| 43.Johansen, M. A. et al 2008 (Norway) | Cross sectional | Diabetic care | Digital fundus photography (Mydriatic, 50^0^, 3F, red-free monochrome) | 20 | Does not involve a proper reference standard | Ophthalmologists | Film fundus photography (Mydriatic, 50^0^, 3F, colour) graded by ophthalmologists | Not mentioned | Not mentioned |
| 44.Kalm, H. et al 1989 (Sweden) | Cross sectional | Primary care | Film fundus photography (Mydriatic, 45^0^, 2F) | 154 | Does not involve digital retinal imaging | ophthalmologist | Dilated slit lamp examination by ophthalmologist | R eye = 87%, L eye = 97% | Not mentioned |
| 45.Kernt, M. et al 2012 (Germany) | Cross sectional | Eye care | Digital fundus photography (Non-mydriatic, 200^0^, 1F, scanning laser) | 141 | Does not involve onsite grading of images, not a study assessing sensitivity/specificity | Independent graders | 7SF ETDRS and dilated slit lamp examination | 7SF – Kappa = 0.79, Slit lamp – Kappa = 0.93 | Not mentioned |
| 46.Kinyoun, J. L. et al 1992 (USA) | Cross sectional | Community based | 7SF ETDRS | 124 | Does not involve digital retinal imaging | Retina Specialist (S) or a Trained Grader (G) | Same images graded by a retina specialist or dilated ophthalmoscopy by a retina specialist (O) | O vs S Kappa = 0.68, O vs G Kappa = 0.49, S vs G Kappa = 0.79 | Not mentioned |
| 47.Klais, C. M. et al 2004 (New Zealand) | Cross sectional | DR screening centre | Digital fundus photography (Mydriatic, 45^0^, 2F) | 1946 | Not a study on DTA. A study on image quality | Retina specialist | Film fundus photography (Mydriatic, 45^0^, 2F) | Not relevant | Not relevant |
| 48.Klein, R. et al 1985 (USA) | Cross sectional | Not mentioned | Film fundus photography (Mydriatic and non-mydriatic, 45^0^, 1F) | 99 | Does not involve digital retinal imaging | Trained graders | Film fundus photography (Mydriatic and non-mydriatic, 30^0^, 3F, stereoscopic) | Not mentioned | Not mentioned |
| 49.Larizza, M. F. et al 2013 (Australia) | Cross sectional | Pathology collection centre | Digital fundus photography (Non-mydriatic, 45^0^, 2F) | 93 | Not a study on DTA. A feasibility study | Trained graders | Not relevant | Not relevant | Not relevant |
| 50.Lau, H. C. et al 1995 (Singapore) | Cross sectional | Primary care | Polaroid fundus photography (Mydriatic, 45^0^) | 13296 | Does not involve digital retinal imaging. Not a study on DTA. | Ophthalmologists | Not relevant | Not relevant | Not relevant |
| 51.Lee, V. S. et al 1993 (USA) | Cross sectional | Not mentioned | Film fundus photography (Mydriatic, 45^0^, 1F) | 410 | Does not involve digital retinal imaging | Reading centre | Dilated indirect ophthalmoscopy by retina specialists | Kappa = 0.74 | Not mentioned |
| 52.Leese, G. P. et al 2002 (UK) | Cross sectional | Not mentioned | Polaroid fundus photography (Non-mydriatic) | 408 | Does not involve digital retinal imaging | Diabetologists | Slit lamp examination by ophthalmologists | Kappa = 0.47 | Not mentioned |
| 53.Liegl, R. et al 2014 (Germany) | Cross sectional | Eye care | Digital fundus photography (Non-mydriatic, 200^0^, 1F, scanning laser) | 143 eyes | Does not involve a proper reference standard | Ophthalmologist | Digital fundus photography (Mydriatic, 45^0^, 2F, stereoscopic) | Kappa = 0.54 | Not mentioned |
| 54.Li, H. K et al 2010 (USA) | Cross sectional | Tertiary level eye clinic | Stereoscopic imaging (Mydriatic, 35, 7F, 35mm) | 85 | Inadequate data for DTA calculations | Image readers | 7F stereoscopic mydriatic 35-degree 35 mm colour slides read by readers | 90 – 100% | 90 – 99% |
| 55.Lin, D. Y. et al 1999 | Literature review | Not relevant | Not relevant | Not relevant | A review article | Not relevant | Not relevant | Not relevant | Not relevant |
| 56.Lin, D. Y. et al 2002 (USA) | Cross sectional | Diabetic care | Digital fundus photography (Non-mydriatic, 45^0^, 1F, monochromatic) | 197 | Does not involve onsite grading of images | Certified reader | 7SF ETDRS and dilated ophthalmoscopy by an ophthalmologist | 78% | 86% |
| 57.Lim, J.L et al 2000 (USA) | Comparative observational case series | University based retina referral practice | Photography with a digital back (N0n-mydriatic, 3F, 45) | 22 | Inadequate data for DTA calculations | Retina specialist | 3F mydriatic 35mm colour slides read by retina specialist | 25 – 100% (Described based on signs) | 90 – 100% (Described based on signs) |
| 58.Liu, F. H. et al 1998 (Taiwan) | Cross sectional | Diabetic care | ? Film fundus photography (Non-mydriatic, 45^0^) | 694 | Does not involve digital retinal imaging | Endocrinologists and ophthalmologist | Not mentioned | 84% | 77% |
| 59.Maberley, D. et al 2004 (Canada) | Cross sectional | Eye care | Digital fundus photography (Mydriatic and non-mydriatic, 45^0^, 1F) | 33 | Not a study on DTA. A study on image quality. | Retina specialist | Same images taken by an experienced professional ophthalmic photographer | Not relevant | Not relevant |
| 60.Marks, J. B. et al 1992 | Review | Not relevant | Not relevant | Not relevant | A review article | Not relevant | Not relevant | Not relevant | Not relevant |
| 61.Martinez, J. et al 2011 (Costa Rica) | Cross sectional | Eye care | Digital fundus photography (Mydriatic and non-mydriatic, 45^0^, 1F) | 1327 | Not a study on DTA. A feasibility study. | Ophthalmologist | Not relevant | Not relevant | Not relevant |
| 62.Milton, R. C. et al 1977 (USA) | Pilot study | Not mentioned | 7SF ETDRS | 148 photos of 22 persons | Does not involve digital retinal imaging. Not a study on DTA. | Ophthalmologist, physician and 2 lay readers | Not relevant | Not relevant | Not relevant |
| 63.Mizrachi, Y. et al 2014 (Israel) | Cross sectional | Primary care | Digital fundus photography (Non-mydriatic, 45^0^, 2F) | 362 | Does not involve onsite grading of images ??? | Retina specialist | Dilated examination by an ophthalmologist | 99.3% | 88.3% |
| 64.Mohan, R. et al 1988 (UK) | Cross sectional | Diabetic care | Polaroid fundus photography (Non-mydriatic, 45^0^, 1F) | 85 | Does not involve digital retinal imaging. | Ophthalmologist | Dilated direct ophthalmoscopy by an ophthalmologist | Not mentioned | Not mentioned |
| 65.Mollentze, W. F. et al 1990 (South Africa) | Cross sectional | Diabetic care | Polaroid fundus photography (Mydriatic and non-mydriatic, 45^0^, 1F) | 86 | Does not involve digital retinal imaging. | Ophthalmologist | Dilated direct ophthalmoscopy | Not mentioned | Not mentioned |
| 66.Moller, F. et al 2001 (Denmark) | Cross sectional | DR screening clinic | Film fundus photography (60^0^, 1F) | 23 | Does not involve digital retinal imaging. | Ophthalmologist | 7SF ETDRS and fluorescein angiography | 88.9% | Not mentioned |
| 67.Moss, S. E. et al 1985 (USA) | Cross sectional | Community based | Direct and indirect ophthalmoscopy | 1949 | Does not involve digital retinal imaging | Ophthalmologist, optometrist and an ophthalmic technician | 7SF ETDRS and 1F  red reflex photograph | Kappa = 0.75 | Not mentioned |
| 68.Moss, S. E. et al 1989 (USA) | Cross sectional | Community based | Film fundus photography (Mydriatic, 2F, 3F, and 4F) | 2694 | Does not involve digital retinal imaging | Reading Centre | 7SF ETDRS | 2F – 87%, 3F – 92%, 4F – 95% | Not mentioned |
| 69.Neubauer, A. S. et al 2008 (Germany) | Randomised controlled trial | Eye care | Digital 7SF ETDRS using Zeiss Visucam PRO NM | 64 | Not a study on DTA for DR screening. A study comparing two cameras. | Reading Centre | Digital 7SF ETDRS using Zeiss FF450plus | 99% | 92% |
| 70.O'Hare, J. P. et al 1996 (UK) | Cross sectional | Primary care | Dilated ophthalmoscopy with and without film fundus photography (Mydriatic, 45^0^, 1F) | 1010 | Does not involve digital retinal imaging | General practitioners or opticians | Dilated ophthalmoscopy and same images graded by an ophthalmologist | Without photo – 70%, With photo – 79% | Without photo – 96%, With photo – 99% |
| 71.Okoli, U. et al 2002 (UK) | Retrospective review | Primary care | Indirect ophthalmoscopy and/or fundus photography | 2230 | Not a study on DTA. Review comparing 3 DR screening models. | General practitioner, orthoptist and optometrists | Not relevant | Not relevant | Not relevant |
| 72.Paton, R. C. et al 1988 | Not relevant | Not relevant | Not relevant | Not relevant | A letter to the editor | Not relevant | Not relevant | Not relevant | Not relevant |
| 73.Penman, A. D. et al 1998 (Egypt) | Cross sectional | Community based | Film fundus photography (Mydriatic, 45^0^, 1F) | 456 | Probably does not involve digital retinal imaging (as study was from 1991-94). Not a study assessing sensitivity/specificity | Reading Centre | Indirect ophthalmoscopy | Kappa = 0.33 | Not mentioned |
| 74.Perez-de-Arcelus, M. et al 2013 | Literature review | Not relevant | Not relevant | Not relevant | A review article | Not relevant | Not relevant | Not relevant | Not relevant |
| 75.Pugh, J. A. et al 1993 (USA) | Cross sectional | Primary care | Film fundus photography (Non-mydriatic 45^0^ 1F monoscopic, and mydriatic 45^0^ 3F mono & stereoscopic) and dilated ophthalmoscopy | 352 | Does not involve digital retinal imaging | Reading centre (RC) & trained internists (TI) for photos, and ophthalmologist (OP) and physician’s assistant (PA) for ophthalmoscopy | 7SF ETDRS | 1F RC – 61%, 1F TI – 54%, 3F RC – 81%, 3F TI – 64%, OP – 33%, PA – 14% | 1F RC – 85%, 1F TI – 87%, 3F RC – 96%, 3F TI – 90%, OP – 99%, PA – 99% |
| 76.Rodriguez Garcia, L. C. et al 2013 (Spain) | Prospective longitudinal descriptive | Primary care | Digital fundus photography | In 2009 – 2850, in 2011 - 3357 | Full article not in English. Not a study on DTA. | ophthalmologist | Not relevant | Not relevant | Not relevant |
| 77.Rogers, D. et al 1990 (UK) | Cross sectional | Primary care | Polaroid fundus photography (Non-mydriatic, 45^0^, 1F) | 84 | Does not involve digital retinal imaging. Not a study on DTA. | General practitioners | Not relevant | Not relevant | Not relevant |
| 78.Romero, P. et al 2010 (Spain) | Audit | Primary care | Digital fundus photography (Mydriatic, 45^0^, 2F) | 879 | An audit. Does not involve a proper reference standard. | Family physicians | Same images graded by an ophthalmologist | 95.2% | 98.6% |
| 79.Ruamviboonsuk, P. et al 2005 (Thailand) | Cross sectional | Diabetic care | Digital fundus photography (Non-mydriatic, 45^0^, 1F) | 150 | Does not involve onsite grading of images | Retina specialist | Dilated fundus examination by retina specialist | 80% | 96% |
| 80.Ruamviboonsuk, P. et al 2006 (Thailand) | Inter-observer reliability study | Various healthcare centres | Digital fundus photography (Mydriatic and non-mydriatic, 45^0^, 1F) | 400 | Does not involve a proper reference standard. A study on inter-observer agreement using images from a database. | Retina specialists (R), ophthalmologists (O), ophthalmic nurses (N) and ophthalmic photographer (P) | Same images graded in consensus by the retina specialists | Median of the 3 values given: R – 93%, O – 86%, N – 89%, P - 86% | Median of the 3 values given: R – 96%, O – 62%, N – 73%, P – 77% |
| 81.Ryder, R. E. et al 1985 (UK) | Cross sectional | Diabetic care | Polaroid fundus photography (Non-mydriatic, 45^0^, 1F) and, dilated & un-dilated ophthalmoscopy | 227 eyes | Does not involve digital retinal imaging. Not a study on DTA. | Not relevant | Not relevant | Not relevant | Not relevant |
| 82.Saari, J. M. et al 2004 (Finland) | Cross sectional | Eye care | Digital fundus photography (Mydriatic, 20^0^ 45^0^, & 50^0^, 2F, colour and 50^0^ 1F red free) | 70 | Does not involve onsite grading of images | Ophthalmologists and a Bachelor of Medicine with special training | Dilated fundus examination by an ophthalmologist in combination with digital colour and red free images | 20^0^ - 6.9%, 45^0^ – 88.9%, 50^0^ colour – 94.0%, 50^0^ red free – 97.7% | 20^0^ – 50.0%, 45^0^ – 100.0%, 50^0^ colour – 99.0%, 50^0^ red free – 98.9% |
| 83.Schwartz, S. et al 2015 (USA) | Cross sectional | DR screening clinics | Digital fundus photography (Non-mydriatic, 45^0^, 1F) | 513 | Not a study on DTA. An evaluation of a DR screening program. | Retina specialists | Not relevant | Not relevant | Not relevant |
| 84.Silva, P. S. et al 2012 (USA) | Instrument  validation study | Eye care | Digital fundus photography (Non-mydriatic, 100^0^ and 200^0^, 1F, stereoscopic) | 103 | Does not involve onsite grading of images | Trained optometrist | 7SF ETDRS | 99% | 100% |
| 85.Soto-Pedre, E. et al 2008 (Spain) | Cross sectional | Diabetic care | Digital fundus photography (Non-mydriatic, 45^0^, 3F) in the eye with the poorer visual acuity | 183 | Does not involve a proper reference standard. | Retina specialist | Same images graded in both eyes | Kappa = 0.75 | Not mentioned |
| 86.Sridhar, G. R. et al 1993 (India) | Cross sectional | Diabetic care | Film fundus photography (Non-mydriatic, 45^0^, 1F) | 42 | Does not involve digital retinal imaging. Not a study on DTA | Not mentioned | Not relevant | Not relevant | Not relevant |
| 87.Tapp, R. J. et al 2015 | Literature review | Not relevant | Not relevant | Not relevant | A review article | Not relevant | Not relevant | Not relevant | Not relevant |
| 88.Taylor, R. et al 1990 (UK) | Cross sectional | Diabetic care | Polaroid fundus photography (Non-mydriatic, 45^0^) and dilated ophthalmoscopy | 2159 | Does not involve digital retinal imaging. Does not involve a proper reference standard. | Photos -consultant physicians, ophthalmoscopy - diabetic clinic doctor | Findings by both methods in combination | Photo – 65.0% , Ophthalmoscopy – 77.5% | Photo - 60.3% , Ophthalmoscopy – 39.7% |

| **Study source** | **Type of Study** | **Setting** | **Screening Strategy** | **Sample size** | **Reason for Exclusion** | **Index Test Grader** | **Reference Standard** | **DTA Values (Sensitivity)** | **DTA Values (Specificity)** |
| --- | --- | --- | --- | --- | --- | --- | --- | --- | --- |
| 89.Van de Kar, W. et al 1990 (Netherlands) | Cross sectional | Primary care | Polaroid fundus photography (1F) | 62 | Does not involve digital retinal imaging. Does not involve a proper reference standard. | General practitioners and hospital physician | Same imaged graded by ophthalmologists | 99% | 55% |
| 90.Von Wendt, G. et al 2000 (Finland) | Cross sectional | Eye care | Film fundus photography (Mydriatic, 60^0^, 1F, colour and red-free black & white) | 74 | Does not involve digital retinal imaging. Does not involve a proper reference standard. | Ophthalmologists | Film fundus photography (Mydriatic, 60^0^, 2F, colour and red-free black & white) | Kappa = 0.84–0.86 | Not mentioned |
| 91.Vujesevic, S. et al 2009 (Italy) | Prospective masked comparative case series | Tertiary level diabetic clinic | Mydriatic retinagraphy device ? (3F, 45 degree) | 55 | Inadequate data for DTA analysis | Retinal specialists | ETDRS 7F 35 mm color slides | 99% | 100% |
| 91.Wareham, N. et al 1991 | Not relevant | Not relevant | Not relevant | Not relevant | An editorial | Not relevant | Not relevant | Not relevant | Not relevant |
| 92.Williams, G. A. et al 2004 | Literature review | Not relevant | Not relevant | Not relevant | A review article | Not relevant | Not relevant | Not relevant | Not relevant |
| 93.Williams, R. et al 1986 (UK) | Cross sectional | Diabetic care | Film or polaroid fundus photography (Non-mydriatic, 45^0^, 1F) | 62 | Does not involve digital retinal imaging. | Ophthalmologists | Dilated fundus examination by an ophthalmologist | 96% | 98% |
| 94.Zafar, A. et al 2008 | Systematic Review | Not relevant | Not relevant | Not relevant | A review article | Not relevant | Not relevant | Not relevant | Not relevant |
| 95.Zhang, X. et al 2007 | Systematic Review | Not relevant | Not relevant | Not relevant | A review article | Not relevant | Not relevant | Not relevant | Not relevant |
