# Supplementary material for: Systematic review and meta-analysis of diagnostic accuracy of detection of any level of diabetic retinopathy using digital retinal imaging
Source: Syst Rev. 2018 Nov 7;7:182. doi: 10.1186/s13643-018-0846-y (PMC6222985; doi:10.1186/s13643-018-0846-y)
Supplement: Supplementary file 3 — Participant characteristics of the included articles. (DOCX 28 kb) [file 13643_2018_846_MOESM3_ESM.docx]

**Additional files 3 –**

**Table 1 - Participants' characteristics of the included studies in the current review -**

Nonmydriatic digital imaging using a single retinal field -

| **Study** | **Country** | **Study Setting** | **Sample Size** | **Mean (SD) Age in Years** | **Percentage of Males** | **Mean (SD) Duration of Diabetes in Years** |
| --- | --- | --- | --- | --- | --- | --- |
| 1.Aptel, F. et al 2008 | France | Not mentioned | 79 | 52.4 (Min:16, Max:89) | 47.1% | 13.6 |
| 2.Baeza, M. et al 2009 | Spain | Primary care | 216 | 68.5 (10.5) | 43.7% | 12.8 (8.9) |
| 3.Ding, J. et al 2012 | China | Primary care | 531 | Min:35, Max:84 | 37.7% | Duration ≤5 in 48.9% |
| 4.Kuo, H. K. et al 2005 | Taiwan | Retinal care | 100 | 59 (Min:31, Max:88) | 61% | Not mentioned |
| 5.Murgatroyd H. et al 2004 | UK | Medical and ophthalmology care | 398 | Median: 63 (Min:17, Max:88) | 57% | 9.3 (8.1) |
| 6.Neubauer, A. S. et al 2008 | Germany | Ophthalmology care | 51 | 60 (12.1) | Not mentioned | 11 (10.1) |
| 7.Phiri, R. et al 2006 | Australia | Retinal and ophthalmology care | 196 | 68.8 (10.1) | 57% | 12.3 (7.7) |
| Scanlon, P. H. et al 2003 (2nd article) | UK | Primary care | 1549 | 65 | Not mentioned | Not mentioned |

Nonmydriatic digital imaging using two retinal fields -

| **Study** | **Country** | **Study Setting** | **Sample Size** | **Mean (SD) Age in Years** | **Percentage of Males** | **Mean (SD) Duration of Diabetes in Years** |
| --- | --- | --- | --- | --- | --- | --- |
| 1.Baeza, M. et al 2009 | Spain | Primary care | 216 | 68.5 (10.5) | 43.7% | 12.8 (8.9) |
| 2.Boucher, M. C. et al 2003 | Canada | Retinal care | 98 | 59.9 (12.2) | 46.9% | Not mentioned |
| 3.Ding, J. et al 2012 | China | Primary care | 531 | Min:35, Max:84 | 37.7% | Duration ≤5 in 48.9% |
| 4.Lopez-Bastida, J. et al 2007 | Spain | Primary care | 773 | Median: 50.8 | 48% | 9.8 (7.1) |

Nonmydriatic digital imaging using >2 fields

| **Study** | **Country** | **Study Setting** | **Sample Size** | **Mean (SD) Age in Years** | **Percentage of Males** | **Mean (SD) Duration of Diabetes in Years** |
| --- | --- | --- | --- | --- | --- | --- |
| 1.Ahmed, J. et al 2006 | USA | Diabetes care | 243 | 60 (11.3) | 54.5% | 8.9 (6.4) |
| 2.Aptel, F. et al 2008 | France | Not mentioned | 79 | 52.4 (Min:16, Max:89) | 47.1% | 13.6 |
| 3.Baeza, M. et al 2009 | Spain | Primary care | 216 | 68.5 (10.5) | 43.7% | 12.8 (8.9) |
| 4.Hansen, A. B. et al 2004 | Denmark | Diabetes care | 83 | 47 (11.2) | 60.2% | 22 (11.8) |
| 5.Hansen, A. B. et al 2004 | Denmark | Diabetes care | 59 | 47 (11.2) | 60.2% | 22 (11.8) |
| 6.Massin, P. et al 2003 | France | Retinal care | 74 | 52 (Min:25, Max:74) | 62.2% | 8 (Min:0, Max:23) |

Mydriatic digital imaging using a single retinal field -

| **Study** | **Country** | **Study Setting** | **Sample Size** | **Mean (SD) Age in Years** | **Percentage of Males** | **Mean (SD) Duration of Diabetes in Years** |
| --- | --- | --- | --- | --- | --- | --- |
| 1.Aptel, F. et al 2008 | France | Not mentioned | 79 | 52.4 (Min:16, Max:89) | 47.1% | 13.6 |
| 2.Baeza, M. et al 2009 | Spain | Primary care | 216 | 68.5 (10.5) | 43.7% | 12.8 (8.9) |
| 3.Ding, J. et al 2012 | China | Primary care | 531 | Min:35, Max:84 | 37.7% | Duration ≤5 in 48.9% |
| 4.Herbert, H. M. et al 2003 | UK | Diabetic retinopathy screening program | 145 | Not mentioned | Not mentioned | Not mentioned |
| 5.Ku, J. J. et al 2013 | Australia | Primary care | 396 | 48 (13) | 36% | Not mentioned |
| 6.Maberley, D. et al 2002 | Canada | Diabetic retinopathy screening program | 100 | 54.6 (13.7) | 31% | Not mentioned |
| 7.Murgatroyd H. et al 2004 | UK | Medical and ophthalmology care | 398 | Median: 63 (Min:17, Max:88) | 57% | 9.3 (8.1) |

Mydriatic digital imaging using two retinal fields -

| **Study** | **Country** | **Study Setting** | **Sample Size** | **Mean (SD) Age in Years** | **Percentage of Males** | **Mean (SD) Duration of Diabetes in Years** |
| --- | --- | --- | --- | --- | --- | --- |
| 1.Baeza, M. et al 2009 | Spain | Primary care | 216 | 68.5 (10.5) | 43.7% | 12.8 (8.9) |
| 2.Ding, J. et al 2012 | China | Primary care | 531 | Min:35, Max:84 | 37.7% | Duration ≤5 in 48.9% |
| 3.Olson, J. A. et al 2003 | UK | Diabetes care | 586 | 56.5 (Min:15.9, Max: 85.4) | 65% | Not mentioned |
| 4.Scanlon, P. H. et al 2003 (1st article) | UK | Retinal care | 239 | Not mentioned | Not mentioned | Not mentioned |
| 5.Scanlon, P. H. et al 2003 (2nd article) | UK | Primary care | 1549 | 65 | Not mentioned | Not mentioned |

Mydriatic digital imaging using >2 retinal fields -

| **Study** | **Country** | **Study Setting** | **Sample Size** | **Mean (SD) Age in Years** | **Percentage of Males** | **Mean (SD) Duration of Diabetes in Years** |
| --- | --- | --- | --- | --- | --- | --- |
| 1.Aptel, F. et al 2008 | France | Not mentioned | 79 | 52.4 (Min:16, Max:89) | 47.1% | 13.6 |
| 2.Baeza, M. et al 2009 | Spain | Primary care | 216 | 68.5 (10.5) | 43.7% | 12.8 (8.9) |
| 3.Hansen, A. B. et al 2004 | Denmark | Diabetes care | 83 | 47 (11.2) | 60.2% | 22 (11.8) |
| 4.Murgatroyd H. et al 2004 | UK | Medical and ophthalmology care | 398 | Median: 63 (Min:17, Max:88) | 57% | 9.3 (8.1) |

DR grading (using digital imaging) by Non-Ophthalmologist HR -

| **Study** | **Country** | **Study Setting** | **Sample Size** | **Mean (SD) Age in Years** | **Percentage of Males** | **Mean (SD) Duration of Diabetes in Years** |
| --- | --- | --- | --- | --- | --- | --- |
| 1.Kuo, H. K. et al 2005 | Taiwan | Retinal care | 100 | 59 (Min:31, Max:88) | 61% | Not mentioned |
| 2.Henricsson, M. et al 2000 | Sweden | Diabetic retinopathy screening program | 283 | Median: 59 (Min:10, Max:84) | 60% | Not mentioned |
| 3.Sundling, V. et al 2013 | Norway | Norwegian Association of Optometry working in private practice | Not mentioned (No of images – DR+ 518, DR- 518) | Not mentioned | Not mentioned | Not mentioned |
| 4.Suansilpong | Thailand | Diabetes care | 248 | 61.1 (10.4) (Min: 30 Max: 83) | 31.9% | (60.5% - <10 years) |

**Table 2 – Participants Characteristics of studies eligible but not included in meta-analysis -**

| **Study** | **Country** | **Study Setting** | **Sample Size** | **Mean (SD) Age in Years** | **Percentage of Males** | **Mean (SD) Duration of Diabetes in Years** |
| --- | --- | --- | --- | --- | --- | --- |
| 1.Bhargava, M. et al 2012 | Singapore | Poly clinics | 397 | 62.9 (11) DR- 65.5(14.9) DR+ | 44% | No DR – 7.3  DR present – 9.9 |
| 2.Mizrachi, Y. et al 2014 | Israel | Community health clinic | 362 | 63.2 | 46.7% | Not mentioned |
| 3. Perrier, M, et al 2003 | Canada | 3ry Retinal clinic | 98 | 59.9  (Range 26 – 92) | 46.9% | Not mentioned |
| 4.Schiffman, R.M. et al 2005 | USA | Retinal Clinic (private sector) | 111 | 57 (14) | 41% | 19 (12) |
| 5.Tu, K.L. et al 2004 | UK | DR screening clinic (Audit) | 126 | 61.2 | 52.1% | Not mentioned |
