# Supplementary material for: Systematic review and meta-analysis of diagnostic accuracy of detection of any level of diabetic retinopathy using digital retinal imaging
Source: Syst Rev. 2018 Nov 7;7:182. doi: 10.1186/s13643-018-0846-y (PMC6222985; doi:10.1186/s13643-018-0846-y)
Supplement: Supplementary file 4 — DTA of different strategies and ungradable image proportions as reported by study authors. (DOCX 29 kb) [file 13643_2018_846_MOESM4_ESM.docx]

**Additional File 4 - DTA of different strategies and ungradable image proportions as reported by study authors**

**Table 1 – Diagnostic test accuracies of identification of any level of diabetic retinopathy using different field strategies, gradability of the images in each study and method of analysis (primary data)**

| **Study** | **Imaging Method** | **Sensitivity (95% CI)** | **Specificity**  **(95% CI)** | **Kappa Statistic for Agreement (95%CI)** | **Grader of Index Test images** | **Reference Test** | **Ungradable Percentage of Tests** | **How ungradable Images were treated by each study authors** | **If analysis was for number of Eyes or Persons** |
| --- | --- | --- | --- | --- | --- | --- | --- | --- | --- |
| **1.Ahmed, J. et al 2006** | Nonmydriatic 3 field | 98% | 100% | N/a | Retina specialist | Dilated funduscopic examination by ophthalmologists (87%) or optometrists  (13%) | 35% | Excluded | Eyes |
| **2.Aptel, F. et al 2008** | Nonmydriatic 1 field | 76.92% | 99.16% | 0.82 | Ophthalmologist | Dilated slit lamp examination by ophthalmologist | 11.4% | Test positive | Eyes |
|  | Nonmydriatic 3 field | 92.31% | 97.48% | 0.90 |  |  | 13.3% |  |  |
|  | Mydriatic  1 field | 89.74% | 98.32% | 0.90 |  |  | 2.5% |  |  |
|  | Mydriatic  3 field | 97.44% | 98.32% | 0.95 |  |  | 3.8% |  |  |
| **3.Baeza, M. et al 2009** | Nonmydriatic  1 field | 68  (60-75)% | 98  (96–100)% | 0.679 | Ophthalmologist | 7SF ETDRS | 15.3% | Not specified (probably test positive) | Not specified (probably persons) |
|  | Nonmydriatic  2 field | 76  (70-83)% | 97  (94-95)% | 0.771 |  |  | 17.1% |  |  |
|  | Nonmydriatic  3 field | 79  (73-86)% | 96  (93-99)% | 0.771 |  |  | 17.6% |  |  |
|  | Mydriatic  1 field | 77  (71-83)% | 98  (96-99)% | 0.767 |  |  | 1.4% |  |  |
|  | Mydriatic  2 field | 86  (81-91)% | 95  (92-98)% | 0.815 |  |  | 1.6 |  |  |
|  | Mydriatic  3 field | 85  (80-90)% | 94  (91-97)% | 0.805 |  |  | 2.1 |  |  |
| **4.Boucher, M. C. et al 2003** | Nonmydriatic  2 field | 95.4 (88.8-98.2)% | 86.4 (77.3- 92.2)% | 0.821  (0.734 -0.907) | Retina specialist | 7SF ETDRS | 12.2% | Excluded | Eyes |
| **5.Ding, J. et al 2012** | Nonmydriatic  1 field | 76.1 (64.4-83.8)% | 80.3 (75.3-84.6)% | N/a | Ophthalmologist | Dilated slit lamp examination by ophthalmologist | 27.1% | Excluded | Persons |
|  | Nonmydriatic  2 field | 90.7 (67.8-84.4)% | 90.7 (67.8-84.4)% | N/a |  |  | 28.2% |  |  |
|  | Mydriatic  1 field | 77.7 (80.8-95.5)% | 76.5 (71.9-80.7)% | N/a |  |  | 8.3% |  |  |
|  | Nonmydriatic  1 field | 85.6 (77.6-91.5)% | 75.6 (70.9-79.9)% | N/a |  |  | 8.9% |  |  |
| **6.Hansen, A. B. et al 2004** | Nonmydriatic  5 field | 96.8% | 85.7% | 0.84  (0.76-0.92) | Retinal Readers | 7SF ETDRS | 7% | Test positive | Persons |
|  | Mydriatic  5 field | 95.2% | 95.2% | 0.88  (0.80-0.96) |  |  | 0% |  |  |
| **7.Henricsson, M. et al 2000** | Mydriatic  3 field | 93% | 91% | 0.77  (0.76 – 0.92) | Ophthalmic Nurse | Same images by Ophthalmologist | 10% | Excluded | Persons |
| **8.Herbert, H. M. et al 2003** | Nonmydriatic (and mydriatic)  1 field | 38.2  (27.6-50.1)% | 95.5  (91.8- 97.5)% | 0.40  (0.27-0.53) | Retina specialist | Dilated slit lamp examination by retina specialist | 4% | Excluded | Eyes |
| **9.Ku, J. J. et al 2013** | Mydriatic  1 field | 74.0  (67.0–80.0)% | 92.0  (90.0 – 94.0)% | 0.67  (0.60 – 0.74) | Ophthalmologist | Dilated slit lamp examination by ophthalmologist | 10.8% | Excluded | Eyes |
| **10.Kuo, H. K. et al 2005** | Nonmydriatic  1 field | 53.8  (43.7-63.6)% | 89.0  (80.9-93.9)% | 0.43  (0.30- 0.55) | Retina specialist | Dilated slit lamp examination by ophthalmologist | 8% | Excluded | Eyes |
| **11.Lopez-Bastida, J. et al 2007** | Nonmydriatic  2 field | 92.0  (90.0-94.0)% | 96.0  (95.0-98.0)% | 0.89 | Retina specialist | Dilated slit lamp examination by retina specialist | 7.2% | Included after making gradable with mydriasis | Persons |
| **12.Maberley, D. et al 2002** | Mydriatic (and nonmydriatic)  1 field | 84.4  (73.4-95.3)% | 79.2  (69.2-89.2)% | 0.62  (0.51- 0.73) | Retina specialist | Dilated slit lamp examination by retina specialist | 0% | Not relevant | Eyes |
| **13.Massin, P. et al 2003** | Nonmydriatic  5 field | 92.0  (86.0- 98.0)% | 88.0  (81- 95)% | N/a | Retina specialist | 7SF ETDRS | 11% | Test positive | Persons |
| **14.Murgatroyd H et al 2003** | Nonmydriatic  1 field | 83.0  (78.0 – 88.0)% | 91.0  (88.0 – 94.0)% | N/a | Retinal readers | Dilated slit lamp examination by ophthalmologist | 26.3% | Excluded | Eyes |
|  | Mydriatic  1 field | 86.0  (82.0-90.0)% | 91.0  (89.0-94.0)% | N/a |  |  | 5.5% |  |  |
|  | Mydriatic  3 field | 90.0  (86.0-93.0)% | 90.0  (88.0-93.0)% | N/a |  |  | 5.3% |  |  |
| **15.Neubauer, A. S. et al 2008** | Nonmydriatic  1 field | 94.0% | 100.0% | 0.68 | Retina specialist | Dilated slit lamp examination by retina specialist | 9.8% | Excluded | Eyes |
| **16.Olson, J. A. et al 2003** | Mydriatic  1 field | 80.0  (74.0- 86.0)% | 88.0  (84.0- 91.0)% | 0.65  (0.58- 0.72) | Trained research registrar | Dilated slit lamp examination by ophthalmologist/ registrar | 3.5% | Excluded | Persons |
|  | Mydriatic  2 field | 83.0  (77.0- 89.0)% | 79.0  (75.0- 83.0)% | 0.56  (0.49- 0.63) |  |  | 4.4% |  |  |
| **17.Phiri, R. et al 2006** | Nonmydriatic  1 field | 86.2  (65.8- 95.3)% | 71.2  (58.1-81.1)% | 0.57  (0.48-0.66) | Retina specialist or Ophthalmologist | 7SF ETDRS | Not given separately for digital images | Excluded | Eyes |
| **18.Scanlon, P. H. et al 2003 (1^st^ article)** | Mydriatic  2 field | 80.2  (75.2-85.2)% | 96.2  (93.2-99.2)% | 0.73 | Specialist Registrar in Ophthalmology | 7SF ETDRS | 1.3% | Excluded | Eyes |
|  |  | 82.8  (78.0-87.6)% | 92.9  (89.6-96.2)% | 0.76 |  | Dilated slit lamp examination by ophthalmologist |  |  |  |
| **19.Scanlon, P. H. et al 2003 (2^nd^ article)** | Nonmydriatic  1 field | 86.0  (80.9-91.1)% | 76.7  (74.5-78.9)% | N/a | Specialist Registrar in Ophthalmology | Dilated slit lamp examination by ophthalmologist | 20.8% | Test positive | Persons |
|  | Mydriatic  2 field | 87.8  (83.0- 92.6)% | 86.1  (84.2- 87.8)% | N/a |  |  | 5.6% |  |  |
| **20.Sundling, V. et al 2013** | Mydriatic  1 field | 67  (62 – 72)% | 84  (80 – 89)% | N/a | Optometrists | Two (100% agreement) ophthalmologists | N/a | Gradable images selected for inclusion | Images |
| **21.Suansilpong, A. et al 2008** | Nonmydriatic  1 field | 65.6  (60.9 – 70.2)% | 84.9  (81.4 – 88.4)% | 0.48 | Endocrinologist | Mydriatic direct and indirect ophthalmoscopy by ophthalmologist | 18.8 | Excluded | Eyes |
