# Supplementary material for: Systematic review and meta-analysis of diagnostic accuracy of detection of any level of diabetic retinopathy using digital retinal imaging
Source: Syst Rev. 2018 Nov 7;7:182. doi: 10.1186/s13643-018-0846-y (PMC6222985; doi:10.1186/s13643-018-0846-y)
Supplement: Supplementary file 5 — DTA parameters by pupil status and field strategy using HSROC curves. (DOCX 20 kb) [file 13643_2018_846_MOESM5_ESM.docx]

**Additional file 5 -** DTA parameters by pupil status and field strategy using HSROC curves

**Table 1.** DTA parameters by pupil status and field strategy using HSROC curves

|  | **Nonmydriatic imaging**  **(1F)** | **Nonmydriatic imaging**  **(2F)** | **Nonmydriatic imaging**  **(>2F)** | **Mydriatic imaging**  **(1F)** | **Mydriatic imaging**  **(2F)** | **Mydriatic imaging**  **(>2F)** |
| --- | --- | --- | --- | --- | --- | --- |
| **Diagnostic odds ratio (DOR) (SE) (95% CI)** | 30.61  (11.7)  (14.4-64.7) | 103.46  (40.6)  (47.8-223.5) | 182.43  (145.2)  (38.3-868.5) | 43.4  (19.7)  (17.8-105.9) | 38.1  (13.2)  (19.3-75.1) | 140  (76.1)  (48.2-406.7) |
| **Sensitivity**  **(SE)**  **(95% CI)** | 75.8%  (3.1)  (69.1-81.4) | 89.9%  (3.0)  (82.1-94.5) | 87.0%  (2.8)  (80.2-91.7) | 77.1%  (5.1)  (65.6-85.6) | 84.3%  (1.1)  (81.9-86.5) | 91.5%  (2.6)  (84.7-95.4) |
| **Specificity**  **(SE)**  **(95% CI)** | 90.7%  (3.4%)  (81.3-95.6) | 92%  (3.0)  (82.1-94.5) | 96.4%  (2.2)  (88.4-98.9) | 92.7%  (2.6)  (85.7-96.4) | 87.5%  (4.2)  (76.5-93.8) | 92.8%  (2.6)  (85.5-96.5) |
| **LR+**  **(positive likelihood ratio)** | 8.2  (2.9)  (4.0-16.5) | 11.2  (4.4)  (5.1-24.5) | 24.5  (15.6)  (7.0-85.8) | 10.6  (3.8)  (5.3-21.5) | 6.7  (2.2)  (3.5-13.1) | 12.7  (4.7)  (6.1-26.4) |
| **LR-**  **(negative likelihood ratio)** | 0.26  (0.03)  (0.2-0.3) | 0.10  (0.03)  (0.06-0.19) | 0.13  (0.03)  (0.08-0.21) | 0.24  (0.05)  (0.15-0.38) | 0.17  (0.01)  (0.15-0.19) | 0.09  (0.02)  (0.04-0.16) |
| **1/LR- (inverse of negative likelihood ratio)** | 3.7  (0.45)  (2.9-4.7) | 9.1  (2.6)  (5.2-16.1) | 7.4  (1.7)  (4.6-11.7) | 4.05  (0.90)  (2.6-6.2) | 5.6  (0.32)  (5.0-6.2) | 11.0  (3.4)  (5.9-20.4) |
|  |  |  |  |  |  |  |
