# Supplementary material for: Systematic review and meta-analysis of diagnostic accuracy of detection of any level of diabetic retinopathy using digital retinal imaging
Source: Syst Rev. 2018 Nov 7;7:182. doi: 10.1186/s13643-018-0846-y (PMC6222985; doi:10.1186/s13643-018-0846-y)
Supplement: Supplementary file 6 — Forest plots of DTA variation by type of reference standard and by the level of service delivery (by clinic settings). (DOCX 536 kb) [file 13643_2018_846_MOESM6_ESM.docx]

**Additional File 6 - Forest plots of DTA variation by type of reference standard and by the level of service delivery (by clinic settings)**


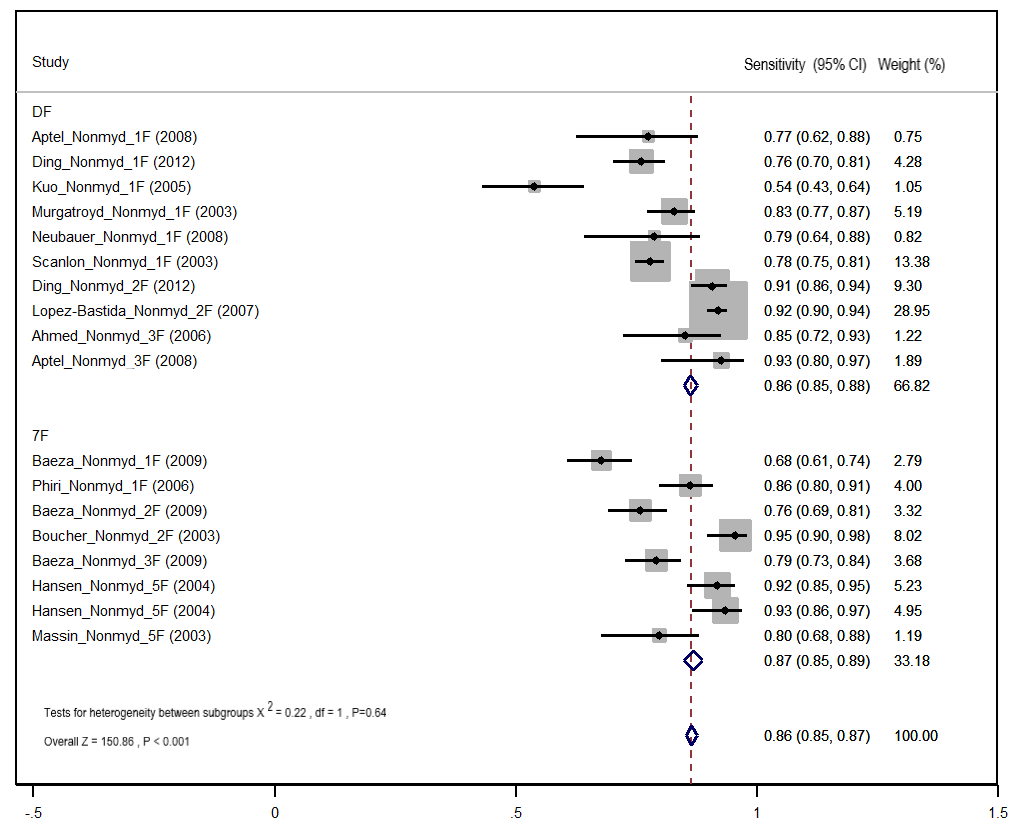


**Figure 1.** Forest plot of summary estimates of sensitivity of non-mydriatic imaging using different reference standards (7F – 7 field ETDRS imaging, DF – mydriatic bio-microscopy/ophthalmoscopy)


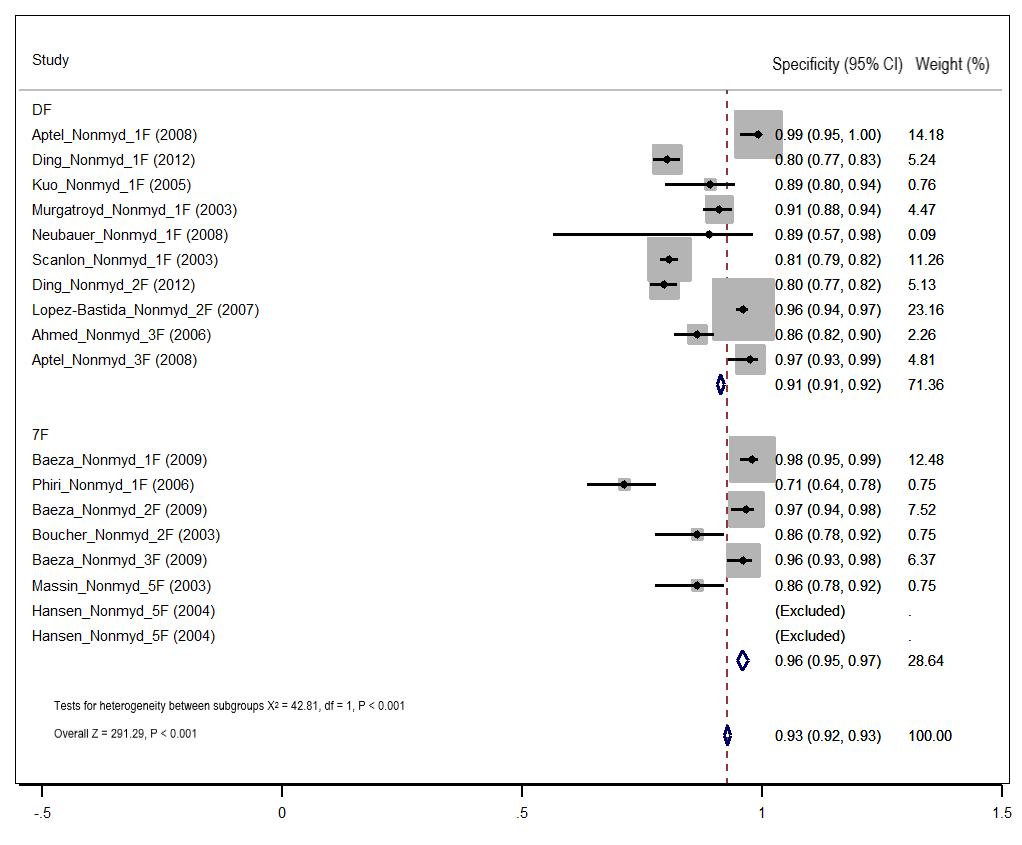


**Figure 2.** Forest plot of summary estimates of specificity of non-mydriatic imaging using different reference standards (7F – 7 field ETDRS imaging, DF – mydriatic bio-microscopy/ophthalmoscopy)


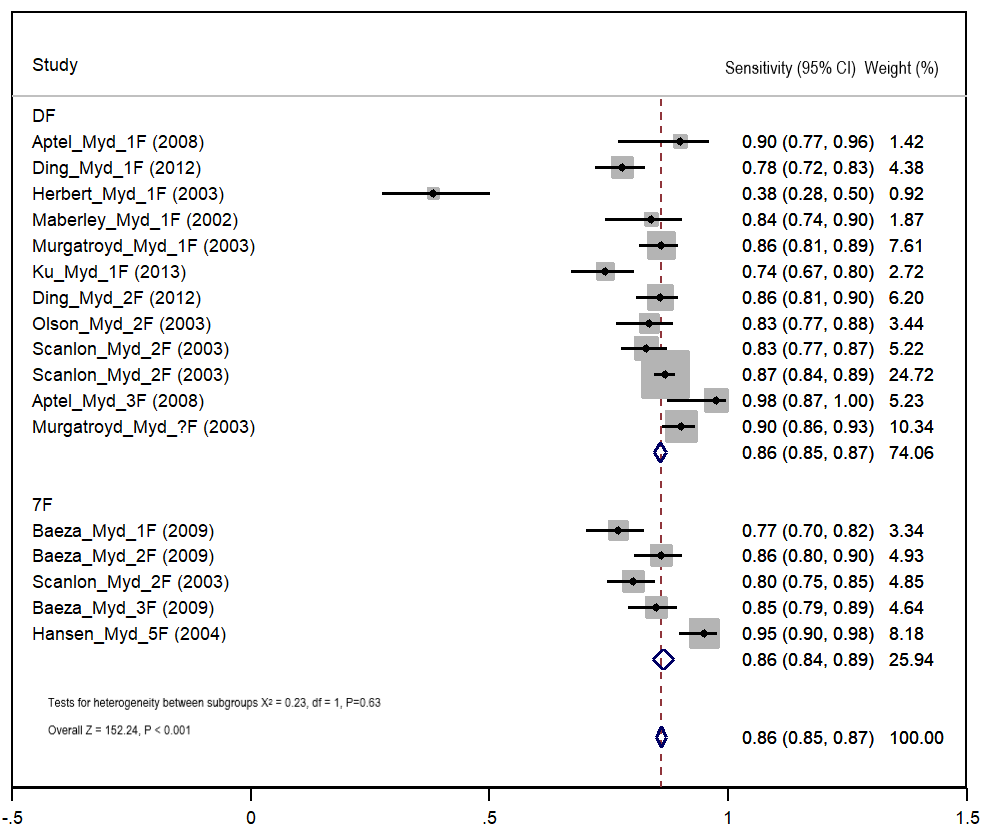


**Figure 3.** Forest plot of summary estimates of sensitivity of mydriatic imaging using different reference standards (7F – 7 field ETDRS imaging, DF – mydriatic bio-microscopy/ophthalmoscopy)


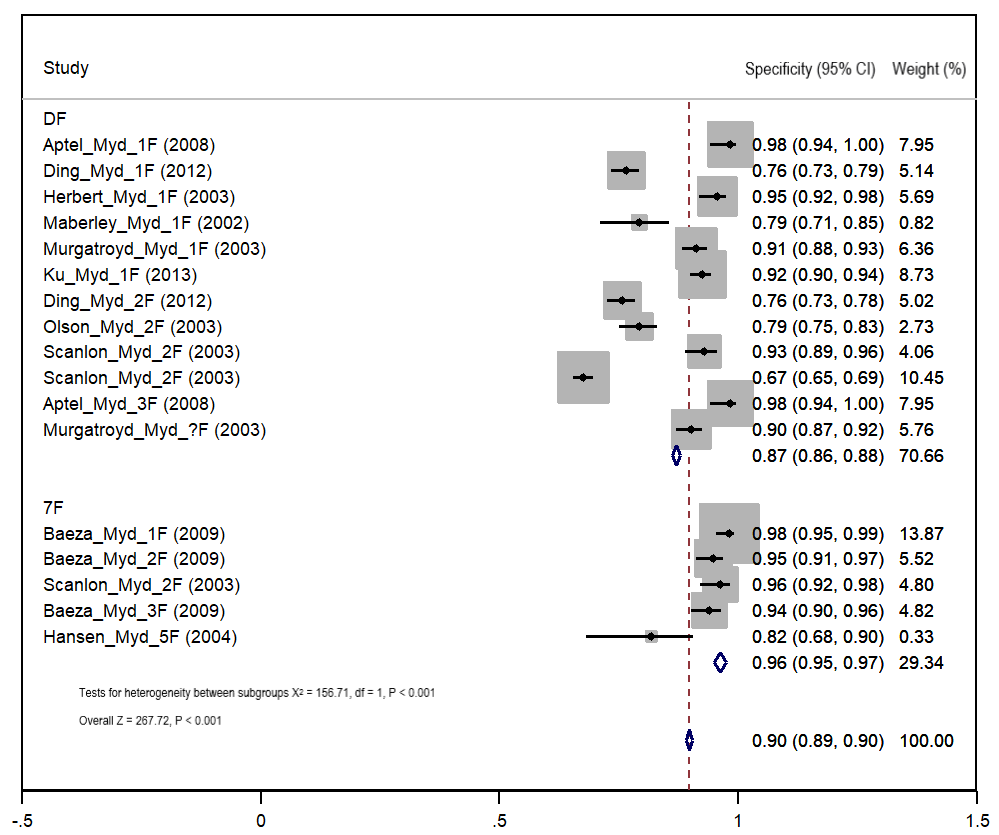


**Figure 4.** Forest plot of summary estimates of specificity of mydriatic imaging using different reference standards (7F – 7 field ETDRS imaging, DF – mydriatic bio-microscopy/ophthalmoscopy)


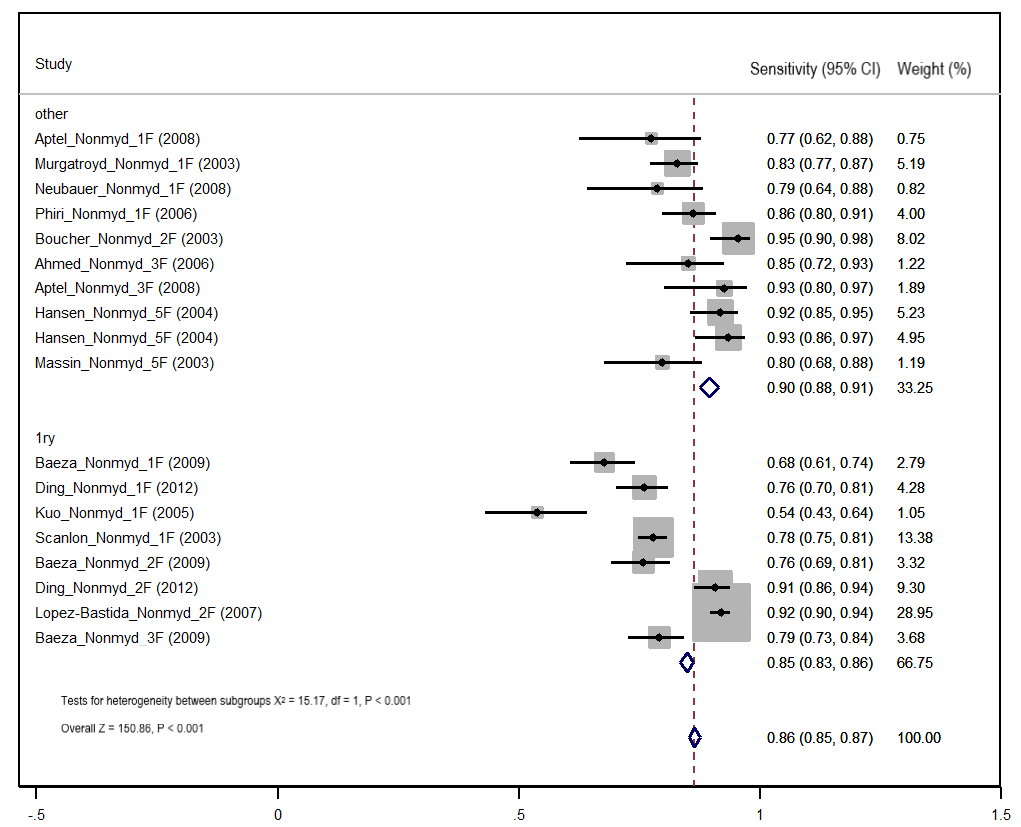


**Figure 5.** Forest plot of summary estimates of sensitivity of non-mydriatic imaging in different settings (Primary – primary level of service delivery, Other - levels other than primary)


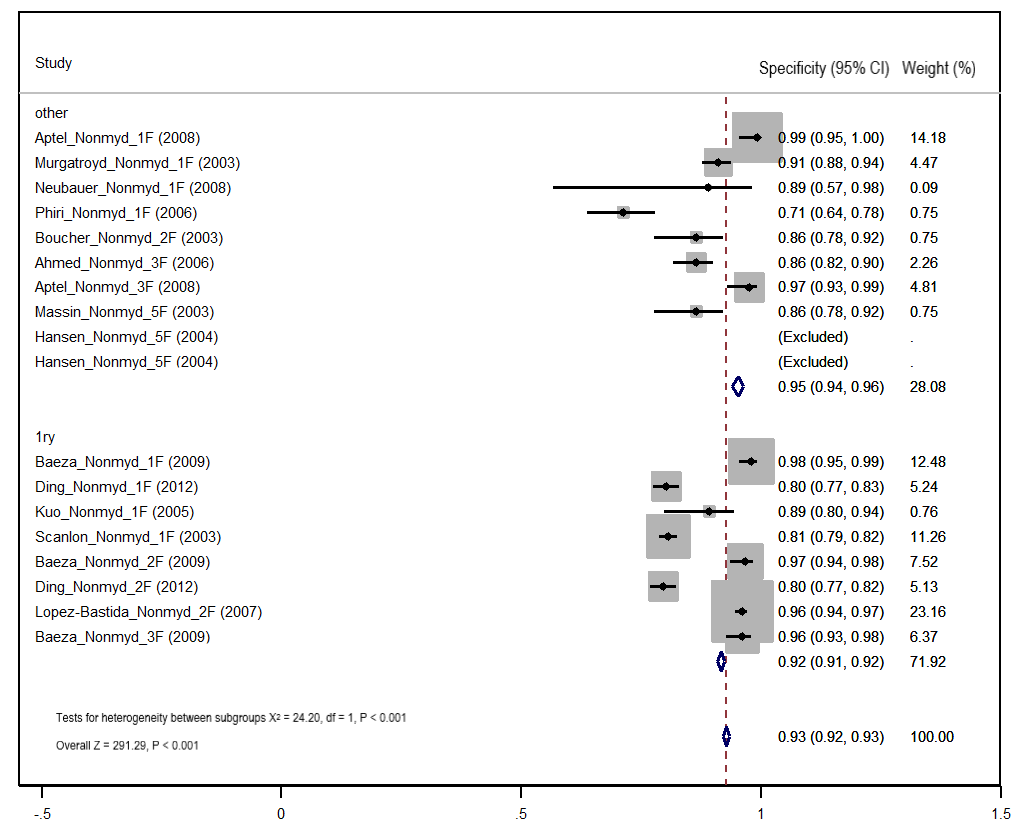


**Figure 6.** Forest plot of summary estimates of specificity of non-mydriatic imaging in different settings (Primary – primary level of service delivery, Other – levels other than primary)


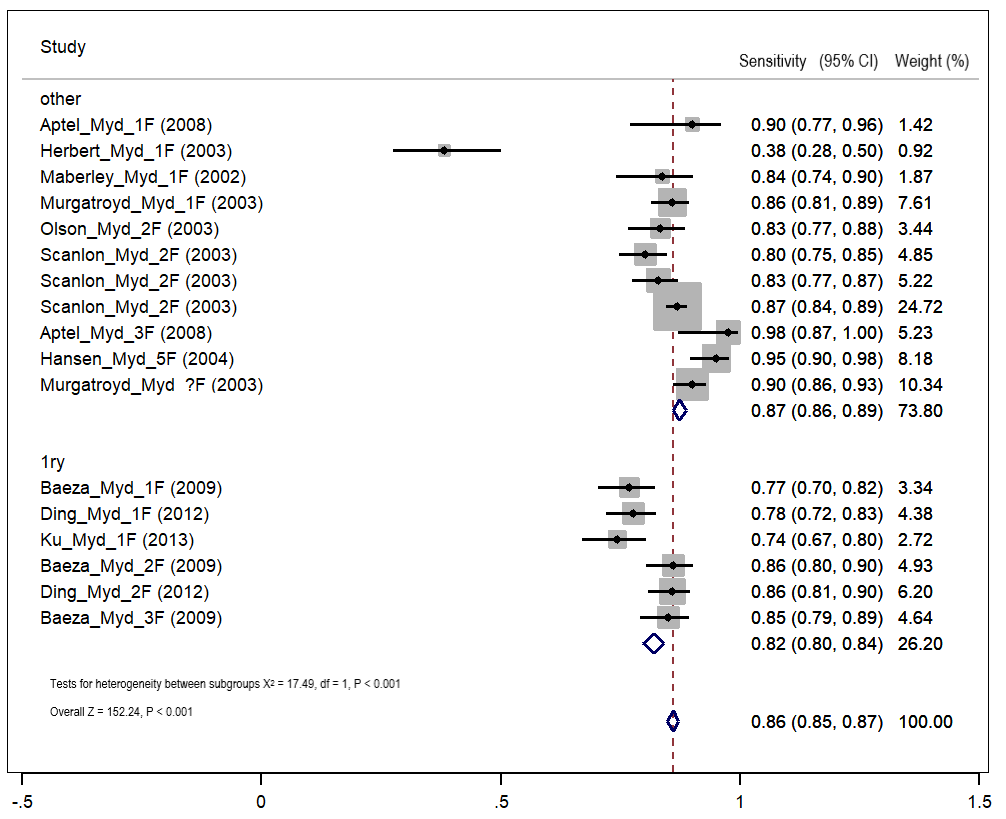


**Figure 7.** Forest plot of summary estimates of sensitivity of mydriatic imaging in different settings (Primary – primary level of service delivery, Other – levels other than primary)


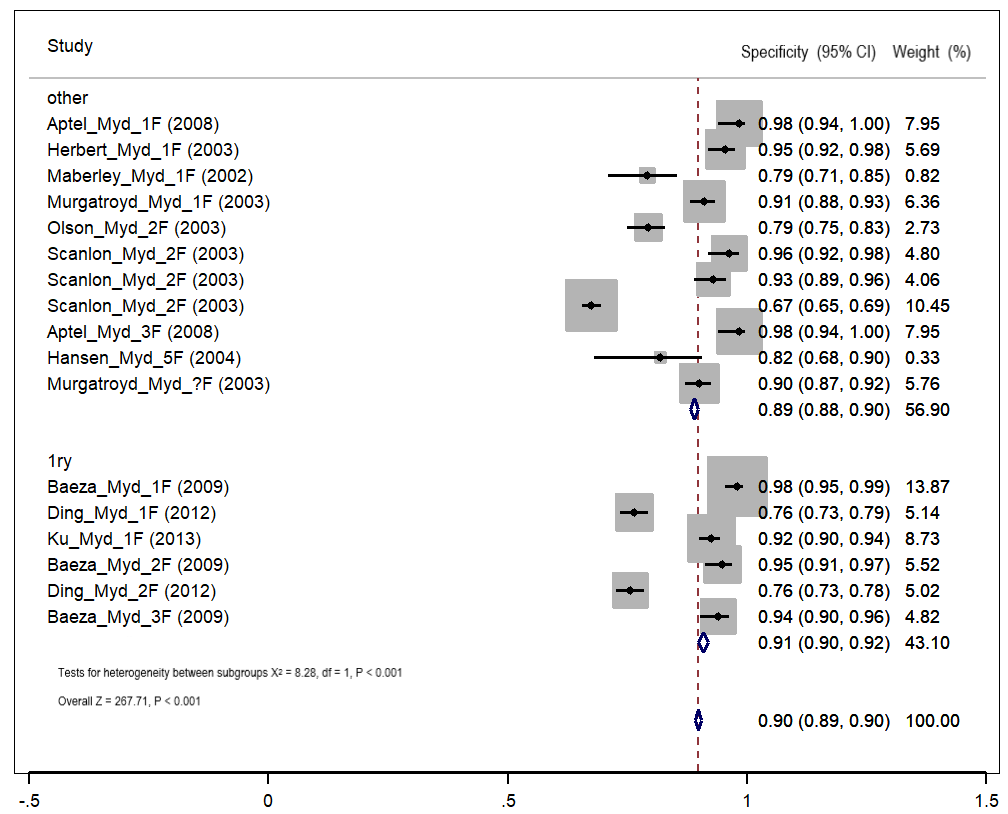


**Figure 8.** Forest plot of summary estimates of specificity of mydriatic imaging in different settings (Primary – primary level of service delivery, Other – levels other than primary)
