# Supplementary material for: Systematic review and meta-analysis of diagnostic accuracy of detection of any level of diabetic retinopathy using digital retinal imaging
Source: Syst Rev. 2018 Nov 7;7:182. doi: 10.1186/s13643-018-0846-y (PMC6222985; doi:10.1186/s13643-018-0846-y)
Supplement: Supplementary file 8 — Forest plots of sub-analyses—DTA using same participant undergoing imaging before and after pupil dilatation. (DOCX 162 kb) [file 13643_2018_846_MOESM8_ESM.docx]

**Additional file 8 – Forest plots of sub-analyses – DTA using same participant undergoing imaging before and after pupil dilatation**


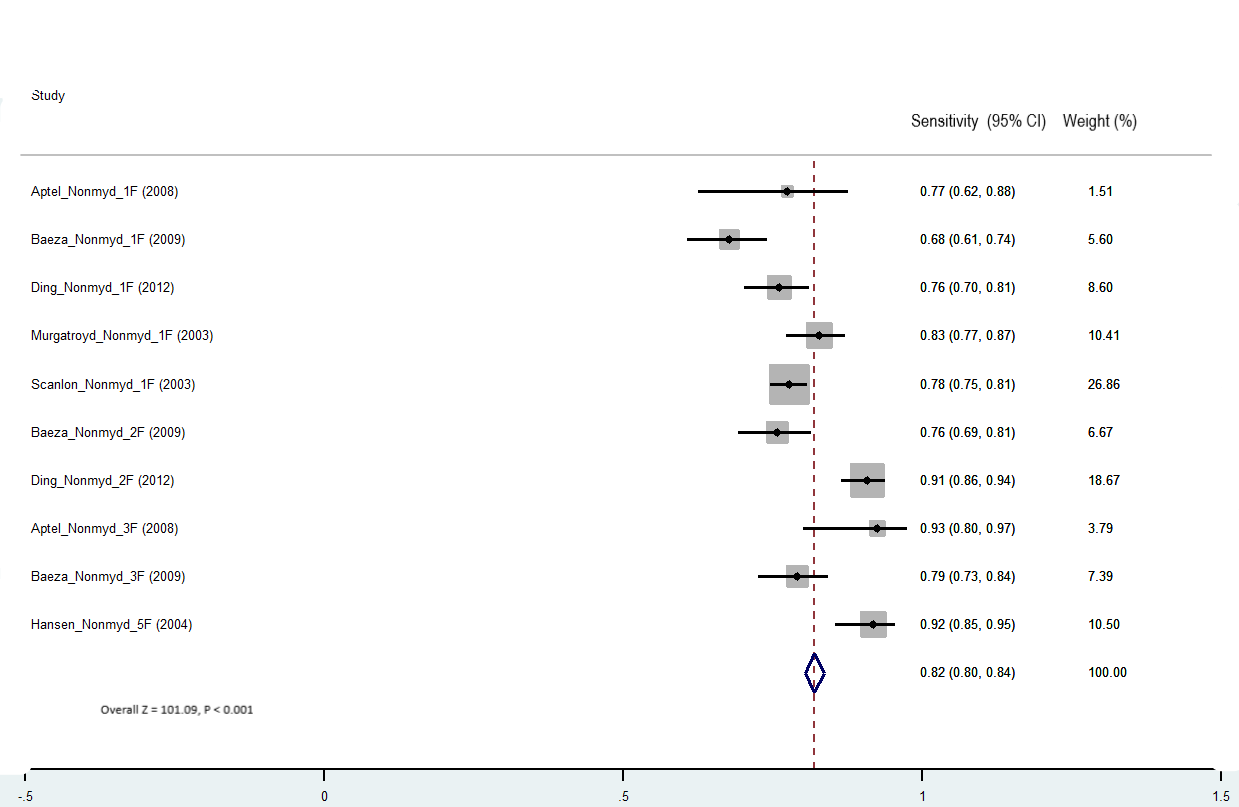


**Figure 1**. Forest plot of summary estimates of sensitivity of non-mydriatic imaging using same participant undergoing imaging before and after pupil dilatation


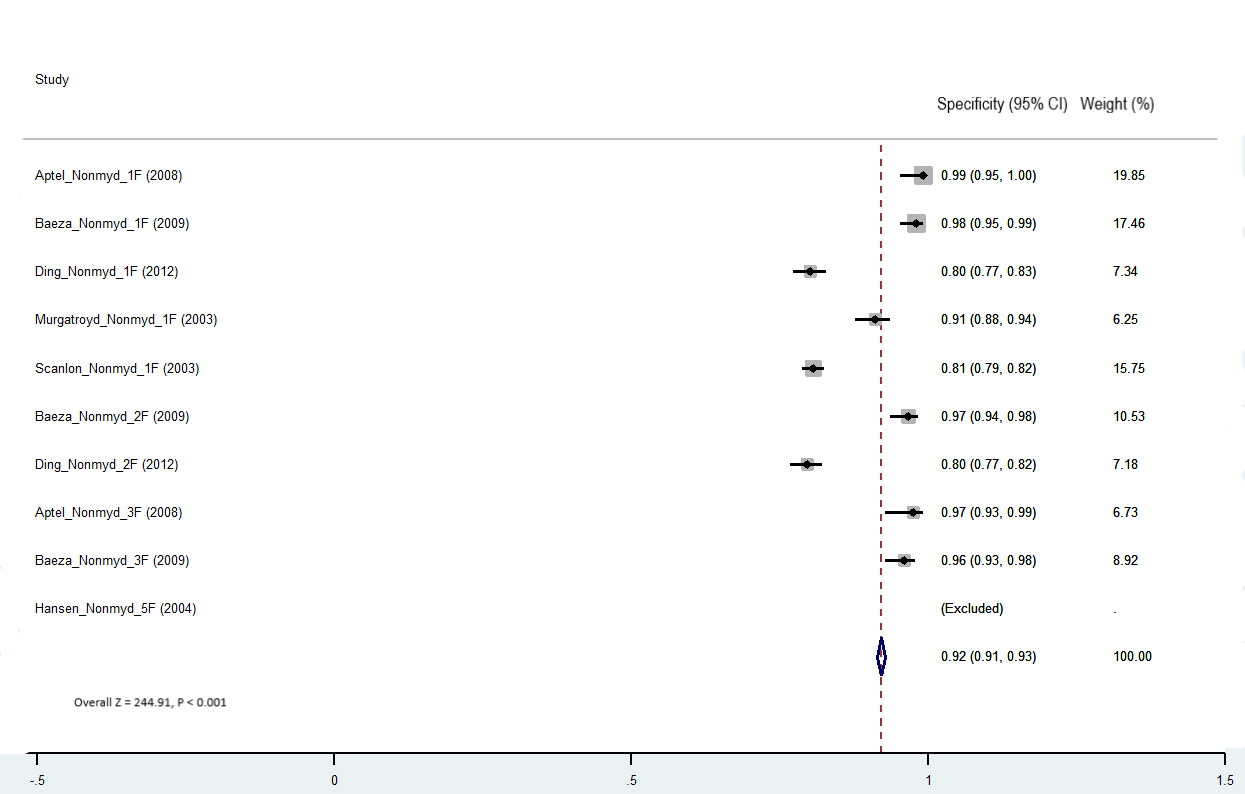


**Figure 2.** Forest plot of summary estimates of specificity of non-mydriatic imaging using same participant undergoing imaging before and after pupil dilatation


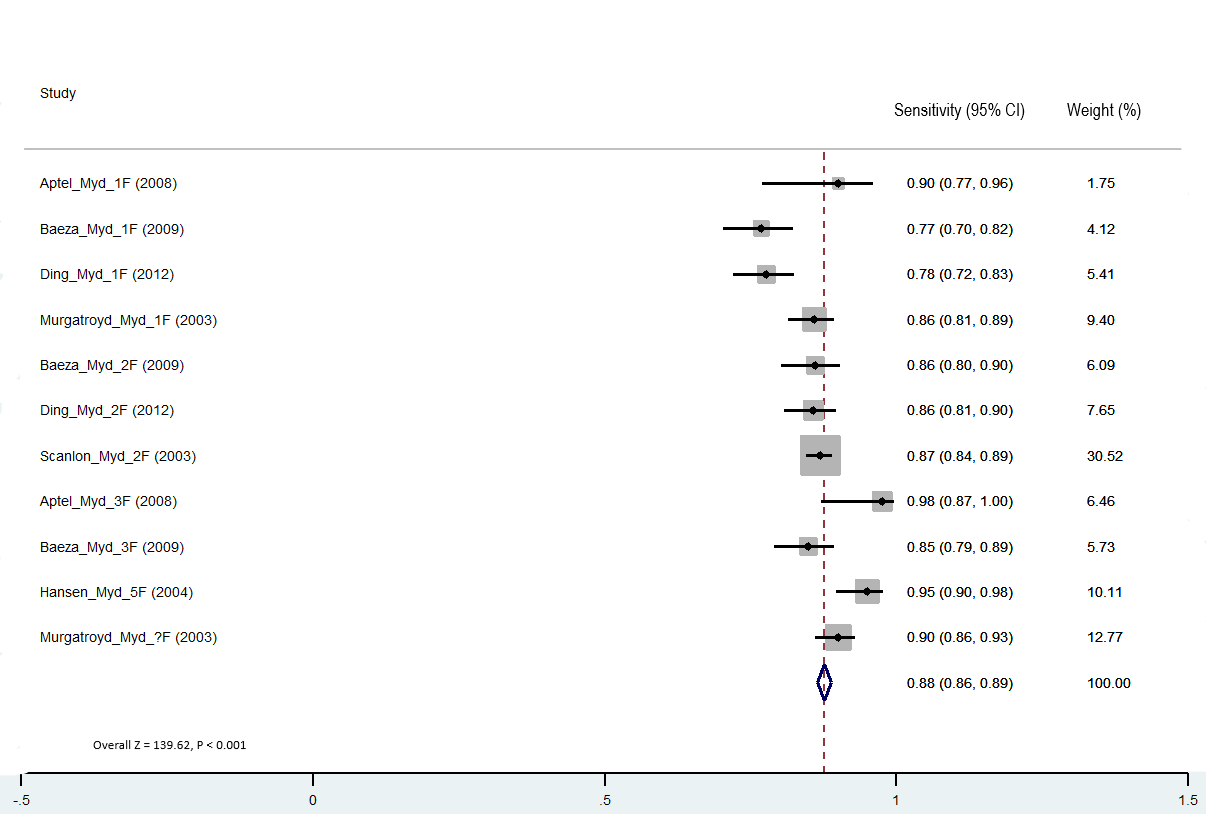


**Figure 3.** Forest plot of summary estimates of sensitivity of mydriatic imaging using same participant undergoing imaging before and after pupil dilatation


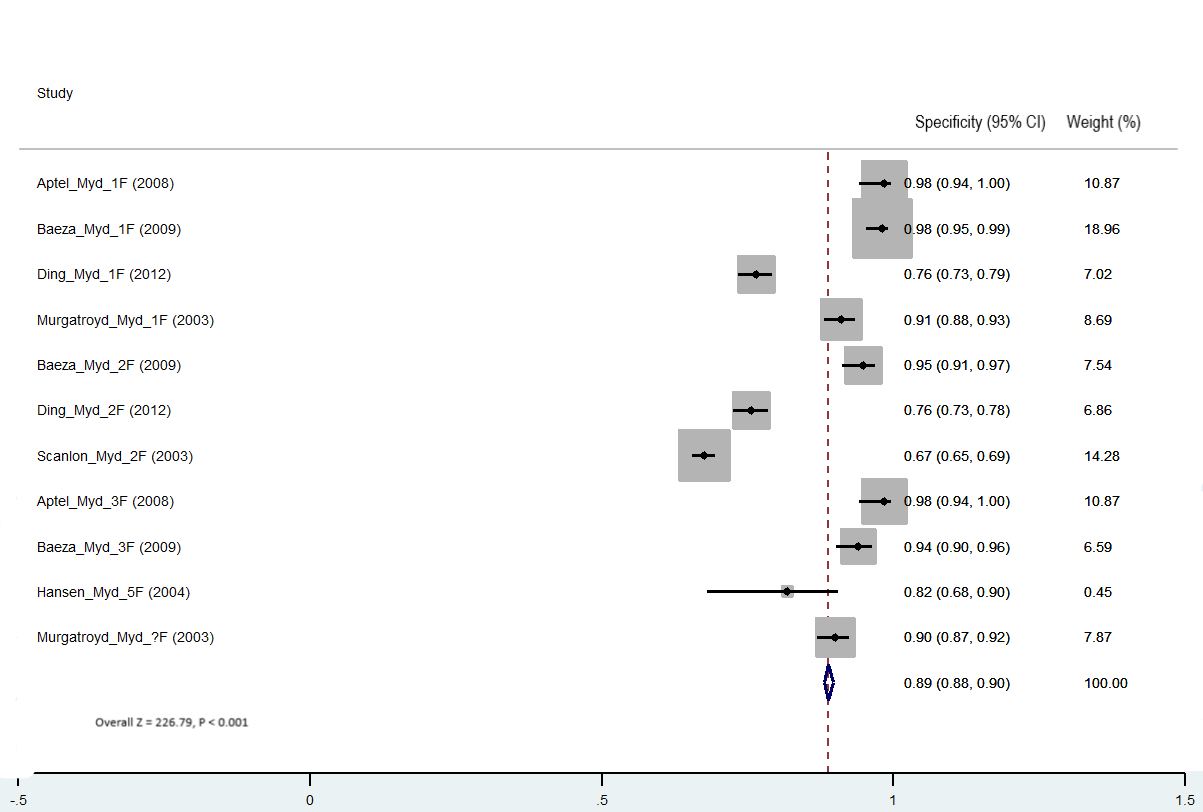


**Figure 4.**  Forest plot of summary estimates of specificity of mydriatic imaging using same participant undergoing imaging before and after pupil dilatation
