# Supplementary material for: Systematic review and meta-analysis of diagnostic accuracy of detection of any level of diabetic retinopathy using digital retinal imaging
Source: Syst Rev. 2018 Nov 7;7:182. doi: 10.1186/s13643-018-0846-y (PMC6222985; doi:10.1186/s13643-018-0846-y)
Supplement: Supplementary file 9 — DTA following adjustments in relevant to exclusion of ungradable proportions in the current review. (DOCX 27 kb) [file 13643_2018_846_MOESM9_ESM.docx]

**Additional File –9 - DTA following adjustments in relevant to exclusion of ungradable proportions in the current review**

**Table 1 – Percentages of ungradable images in each strategy, how it was treated in DTA calculations and adjusted DTA based on proportions of un-gradable images reported –**

| **Study** | **Imaging strategy (No: of fields and pupillary status)** | **Sensitivity (95% CI)** | **Specificity (95% CI)** | **Kappa (inter grader agreement) (95%CI)** | **Grader of Index Test images** | **Reference Test** | **Ungradable Percentage of Tests** | **How ungradable Images were treated by review authors** | **If analysis was for number of Eyes or Persons** |
| --- | --- | --- | --- | --- | --- | --- | --- | --- | --- |
| **Ahmed, J. et al 2006** | Nonmydriatic 3 field | 85.71% (73.33- 92.90)% | 86.64% (81.99- 90.24)% | 0.587  (0.478-0.696) | Retina specialist | Dilated funduscopic examination by ophthalmologists (87%) or optometrists  (13%) | 35% | Excluded | Eyes |
| **Aptel, F. et al 2008** | Nonmydriatic 1 field | 76.92% | 99.16% | 0.82 | Ophthalmologist | Dilated slit lamp examination by ophthalmologist | 11.4% | Test positive **(unable to calculate values excluding ungradables with the given data)* | Eyes |
|  | Nonmydriatic 3 field | 92.31% | 97.48% | 0.90 |  |  | 13.3% |  |  |
|  | Mydriatic 1 field | 89.74% | 98.32% | 0.90 |  |  | 2.5% |  |  |
|  | Mydriatic 3 field | 97.44% | 98.32% | 0.95 |  |  | 3.8% |  |  |
| **Baeza, M. et al 2009** | Nonmydriatic 1 field | 68  (60-75)% | 98  (96–100)% | 0.679 | Ophthalmologist | 7SF ETDRS | 15.3% | Not specified **(probably test positive) (unable to calculate values excluding ungradables with the given data)* | Not specified (probably persons) |
|  | Nonmydriatic 2 field | 76%  (70-83)% | 97 (94-95)% | 0.771 |  |  | 17.1% |  |  |
|  | Nonmydriatic 3 field | 79 %  (73-86)% | 96 (93-99)% | 0.771 |  |  | 17.6% |  |  |
|  | Mydriatic 1 field | 77%  (71-83)% | 98 (96-99)% | 0.767 |  |  | 1.4% |  |  |
|  | Mydriatic 2 field | 86%  (81-91)% | 95 (92-98)% | 0.815 |  |  | 1.6 |  |  |
|  | Mydriatic 3 field | 85%  (80-90)% | 94 (91-97)% | 0.805 |  |  | 2.1 |  |  |
| **Boucher, M. C. et al 2003** | Nonmydriatic 2 field | 95.4%  (88.8-98.2)% | 86.4%  (77.3- 92.2)% | 0.821 (0.734 -0.907) | Retina specialist | 7SF ETDRS | 12.2% | Excluded | Eyes |
| **Ding, J. et al 2012** | Nonmydriatic 1 field | 76.1%  (64.4-83.8)% | 80.3% (75.3-84.6)% | - | Ophthalmologist | Dilated slit lamp examination by ophthalmologist | 27.1% | Excluded | Persons |
|  | Nonmydriatic 2 field | 90.7% (67.8-84.4)% | 90.7% (67.8-84.4)% | - |  |  | 28.2% |  |  |
|  | Mydriatic 1 field | 77.7%  (80.8-95.5)% | 76.5% (71.9-80.7)% | - |  |  | 8.3% |  |  |
|  | Nonmydriatic 1 field | 85.6% (77.6-91.5)% | 75.6% (70.9-79.9)% | - |  |  | 8.9% |  |  |
| **Hansen, A. B. et al 2004** | Nonmydriatic 5 field | 92.50% (86.36-96.00)% | 100.00% (89.85-100.00)% | 0.84 (0.75-0.94) | Retinal Readers | 7SF ETDRS | 7% | Excluded | Eyes |
|  | Mydriatic 5 field | 93.80% (88.24- 96.82)% | 100.00 %(90.36- 100.00)% | 0.87 (0.78-0.96) |  |  | 0% |  |  |
| **Herbert, H. M. et al 2003** | Nonmydriatic (and mydriatic) 1 field | 38.2% (27.6-50.1)% | 95.5% (91.8- 97.5)% | 0.40 (0.27-0.53) | Retina specialist | Dilated slit lamp examination by retina specialist | 4% | Excluded | Eyes |
| **Ku, J. J. et al 2013** | Mydriatic 1 field | 74.0% (67.0–80.0)% | 92.0% (90.0–  94.0)% | 0.67 (0.60-  0.74) | Ophthalmologist | Dilated slit lamp examination by ophthalmologist | 10.8% | Excluded | Eyes |
| **Kuo, H. K. et al 2005** | Nonmydriatic 1 field | 53.8% (43.7-63.6)% | 89.0% (80.9-93.9)% | 0.43 (0.30- 0.55) | Retina specialist | Dilated slit lamp examination by ophthalmologist | 8% | Excluded | Eyes |
| **Lopez-Bastida, J. et al 2007** | Nonmydriatic 2 field | 92.0% (90.0-94.0)% | 96.0% (95.0-98.0)% | 0.89 | Retina specialist | Dilated slit lamp examination by retina specialist | 7.2% | Excluded *(*Included after making gradable with mydriasis)* | Persons |
| **Maberley, D. et al 2002** | Mydriatic (and nonmydriatic) 1 field | 84.4% (73.4-95.3)% | 79.2% (69.2-89.2)% | 0.62 (0.51- 0.73) | Retina specialist | Dilated slit lamp examination by retina specialist | 0% | Not relevant | Eyes |
| **Massin, P. et al 2003** | Nonmydriatic 5 field | 80.85% (67.46-89.58)% | 86.59%  (77.55-92.34)% | 0.67 (0.53-0.80) | Retina specialist | 7SF ETDRS | 11% | Excluded | Eyes |
| **Murgatroyd H et al 2003** | Nonmydriatic 1 field | 83.0%  (78.0 - 88.0)% | 91.0%  (88.0 - 94.0)% | - | Retinal readers | Dilated slit lamp examination by ophthalmologist | 26.3% | Excluded | Eyes |
|  | Mydriatic 1 field | 86.0% (82.0-90.0)% | 91.0% (89.0-94.0)% | - |  |  | 5.5% |  |  |
|  | Mydriatic 3 field | 90.0%  (86.0-93.0)% | 90.0% (88.0-93.0)% | - |  |  | 5.3% |  |  |
| **Neubauer, A. S. et al 2008** | Nonmydriatic 1 field | 77.78% (45.26-93.68)% | 94.59% (82.30-98.50)% | 0.72 (0.47-0.98) | Retina specialist | Dilated slit lamp examination by retina specialist | 9.8% | Excluded | Eyes |
| **Olson, J. A. et al 2003** | Mydriatic 1 field | 80.0% (74.0- 86.0)% | 88.0% (84.0- 91.0)% | 0.65 (0.58- 0.72) | Trained research registrar | Dilated slit lamp examination by ophthalmologist/ registrar | 3.5% | Excluded | Persons |
|  | Mydriatic 2 field | 83.0% (77.0- 89.0)% | 79.0% (75.0- 83.0)% | 0.56 (0.49- 0.63) |  |  | 4.4% |  |  |
| **Phiri, R. et al 2006** | Nonmydriatic 1 field | 86.2% (65.8- 95.3)% | 71.2% (58.1-81.1)% | 0.57 (0.48-0.66) | Retina specialist or Ophthalmologist | 7SF ETDRS | Not given separately for digital images | Excluded | Eyes |
| **Scanlon, P. H. et al 2003 (1st article)** | Mydriatic 2 field | 80.2% (75.2-85.2)% | 96.2% (93.2-99.2)% | 0.73 | Specialist Registrar in Ophthalmology | 7SF ETDRS | 1.3% | Excluded | Eyes |
|  |  | 82.8% (78.0-87.6)% | 92.9% (89.6-96.2)% | 0.76 |  | Dilated slit lamp examination by ophthalmologist |  |  |  |
| **Scanlon, P. H. et al 2003 (2nd article)** | Nonmydriatic 1 field | 77.84% ( 73.21-81.87)% | 80.67% (77.91- 83.16)% | 0.54 (0.49-0.59) | Specialist Registrar in Ophthalmology | Dilated slit lamp examination by ophthalmologist | 20.8% | Excluded | Persons |
|  | Mydriatic 2 field | 86.88% (83.41-89.71)% | 67.48% (64.55-70.29)% | 0.46 (0.42-0.50) |  |  | 5.6% |  |  |
